# Experimental Validation of Model-Based Prognostics for Pneumatic Valves

**Published:** 2018-01-19

**Authors:** Chetan S. Kulkarni, Matthew J. Daigle, George Gorospe, Kai Goebel

**Affiliations:** 1 SGT, Inc., NASA Ames Research Center, Moffett Field, CA, 94035, USA; 2 NASA Ames Research Center, Moffett Field, CA, 94035, USA

## Abstract

Because valves control many critical operations, they are prime candidates for deployment of prognostic algorithms. But, similar to the situation with most other components, examples of failures experienced in the field are hard to come by. This lack of data impacts the ability to test and validate prognostic algorithms. A solution sometimes employed to overcome this shortcoming is to perform run-to-failure experiments in a lab. However, the mean time to failure of valves is typically very high (possibly lasting decades), preventing evaluation within a reasonable time frame. Therefore, a mechanism to observe development of fault signatures considerably faster is sought. Described here is a testbed that addresses these issues by allowing the physical injection of leakage faults (which are the most common fault mode) into pneumatic valves. What makes this testbed stand out is the ability to modulate the magnitude of the fault almost arbitrarily fast. With that, the performance of end-of-life estimation algorithms can be tested. Further, the testbed is mobile and can be connected to valves in the field. This mobility helps to bring the overall process of prognostic algorithm development for this valve a step closer to validation. The paper illustrates the development of a model-based prognostic approach that uses data from the testbed for partial validation.

## 1. Introduction

Valves, and pneumatically-actuated valves in particular, play a critical role in many systems, in cryogenic propellant loading systems for controlling the flow of propellant (Daigle & Goebel, 2011), in aircraft carrier steam catapults (Shevach et al., 2014), the residual heat removal system in a nuclear power plant (Lin, Li, & Zio, 2014), and air bleed systems in aircraft (Lorton, Fouladirad, & Grall, 2013). In these kinds of systems, valve failures can have an adverse impact on system safety and availability. Hence, there is a critical need for valve health monitoring and failure prediction, and to develop prognostic methods for computing end of life (EOL) and remaining useful life (RUL).

Experimental testbeds play a key role in maturing prognostics algorithms. In particular, such testbeds require repeatable and controllable injection of faults and degradations. Such testbeds have been constructed for electrical power systems (Poll, Patterson-Hine, Camisa, Garcia, et al., 2007; Poll, Patterson-Hine, Camisa, Nishikawa, et al., 2007), electromechanical actuators (Balaban et al., 2010), mobile robots (Tang, Hettler, Zhang, & DeCastro, 2011; Balaban et al., 2013) and fuel systems (Niculita, Jennions, & Irving, 2013). For the purpose of maturing and validating valve prognostics approaches, a pneumatic valve testbed was developed as discussed in (Kulkarni, Daigle, & Goebel, 2013).

The contributions of this work are twofold. In the first phase a hardware-in-the-loop testbed was developed for pneumatic valves used for cryogenic propellant loading operations. The testbed allows four different kinds of leakage faults to be injected and their magnitude controlled to any desired fault progression function. The setup is similar to the one with actual propellant loading systems in the field. The approach is extended to enable prognostics in real time, and demonstrated using real data from the pneumatic valve testbed.

In the second phase a model-based prognosis framework is implemented to two types of pneumatic valves. Unlike earlier work based on particle filters (Daigle & Goebel, 2011), in this paper a new model-based method using measurements of valve open and close times is discussed, recently developed in (Daigle, Kulkarni, & Gorospe, 2014). In real valve operations, typically only valve position is measured, from which the only meaningful information for prognostics are the valve open and close times. Valve open/close times are computed based on defined fully open/close position thresholds which are defined based on respective valve operation. Difference between completely open and completely close positions is measured to compute time. Given that the valves are operated discretely, and we measure position, we can just compute the time difference of when these positions are measured. No ”air leakage” measurement sensor is available in the field, hence this is inferred in order to perform prognostics. While the information is sparse compared to an environment with rich sensor information. The overall model is simpler and requires significantly less computation to isolate and identify faults, and predict EOL and RUL. However, tradeoffs are made with regards to prognostic horizon. The approach follows the general estimation-prediction framework for model-based prognostics (Orchard & Vachtsevanos, 2009; Daigle & Goebel, 2013).

The paper is organized as follows. Section 2 describes the valve prognostics testbed. Section 3 explains the valve models. Section 4 provides the valve prognosis framework, and Section 5 presents prognosis results using testbed data. Section 6 discusses related work. Section 7 concludes the paper.

## 2. Valve Testbed

**Figure 1.**
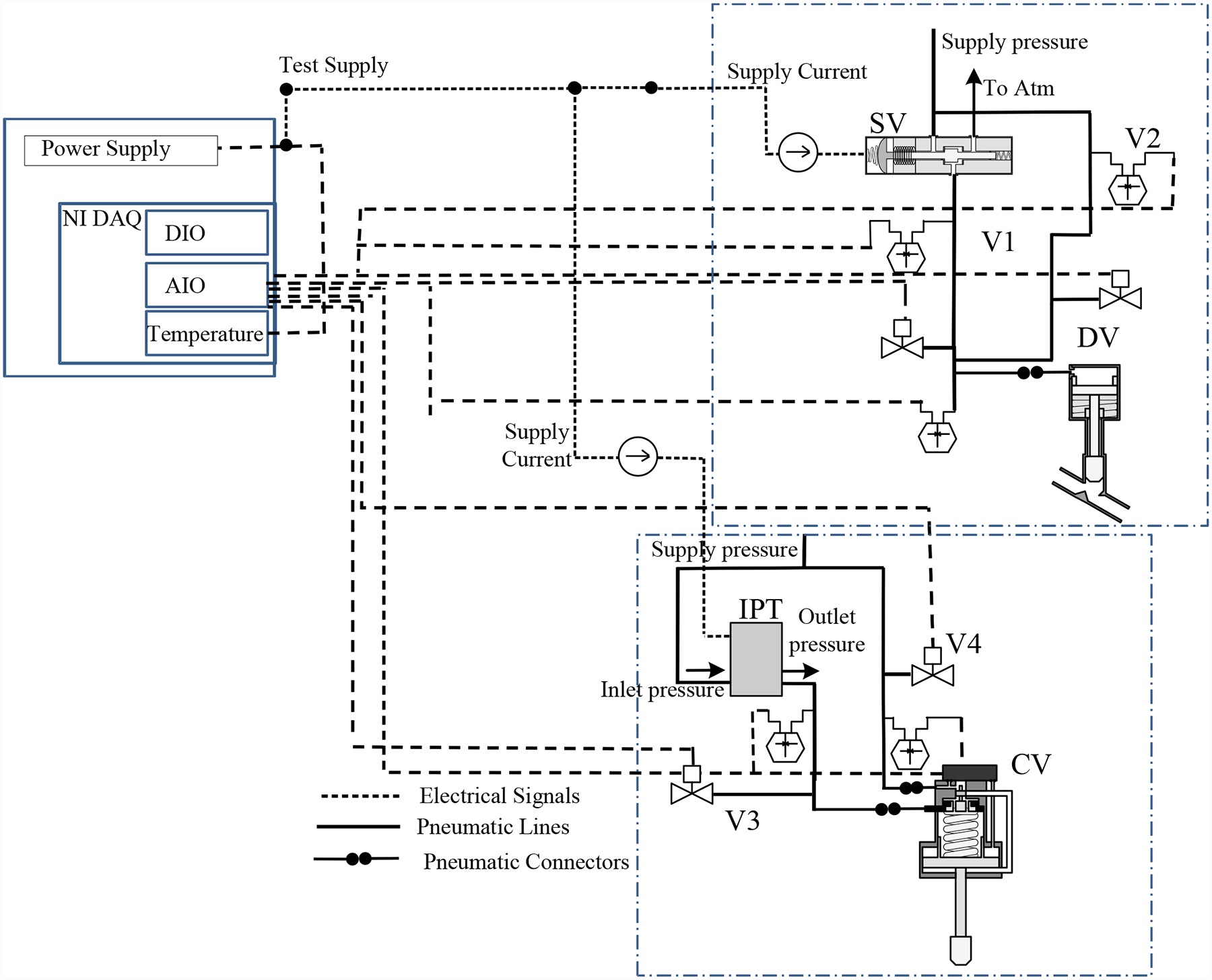
Prognostics demonstration testbed schematic.

**Figure 2.**
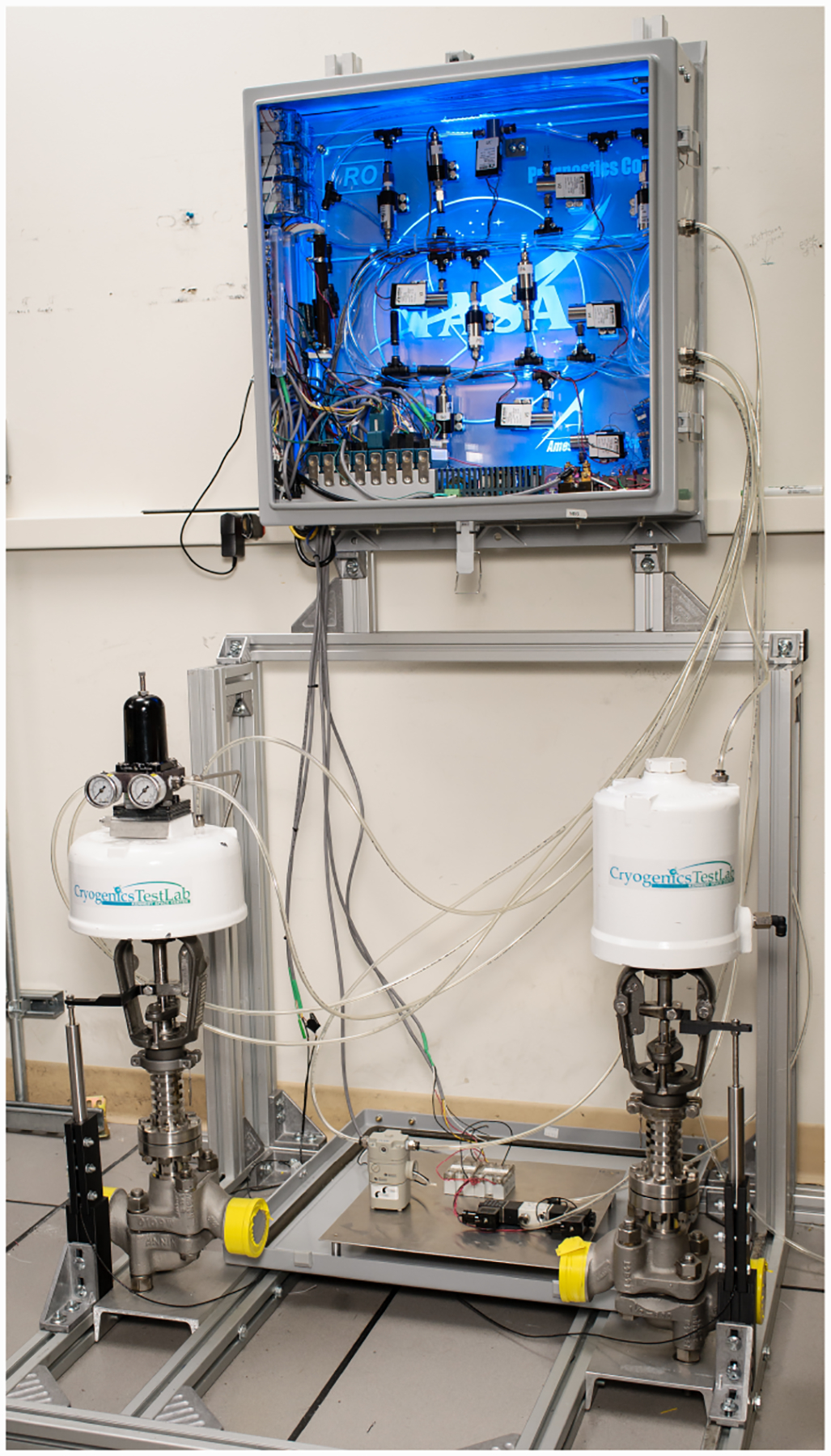
Developed Testbed

The valve prognostics testbed, schematic shown in Fig. 1 and actual developed testbed shown in Fig. 2, has been developed to demonstrate valve prognosis in the context of cryogenic refueling operations (Kulkarni et al., 2013). The dashed lines denote the electrical signals, including the data acquisition I/O signals, power lines, etc. The solid lines denote the pneumatic pressure lines connecting the supply and the valves. Electric power is routed through a power supply that has a fail-safe mode which in turn isolates the valve prognostics testbed from the field cryogenic loading system interface in case of an emergency.

The testbed contains a discretely-controlled valve (DV), a solenoid valve (SV), a continuously-controlled valve (CV), a current-pressure transducer (IPT), and a number of proportional valves for injecting leakage faults. The components are described in the following subsections.

The fault injection testbed is portable. That is, it can be moved from the lab environment and it can be connected to the actual propellent loading system on field. This gives the testbed the unique ability to test faults on any of the discrete and continuously-controlled valves not only during development in the lab but also for validation in the production environment.

### 2.1. DV Operation

**Figure 3.**
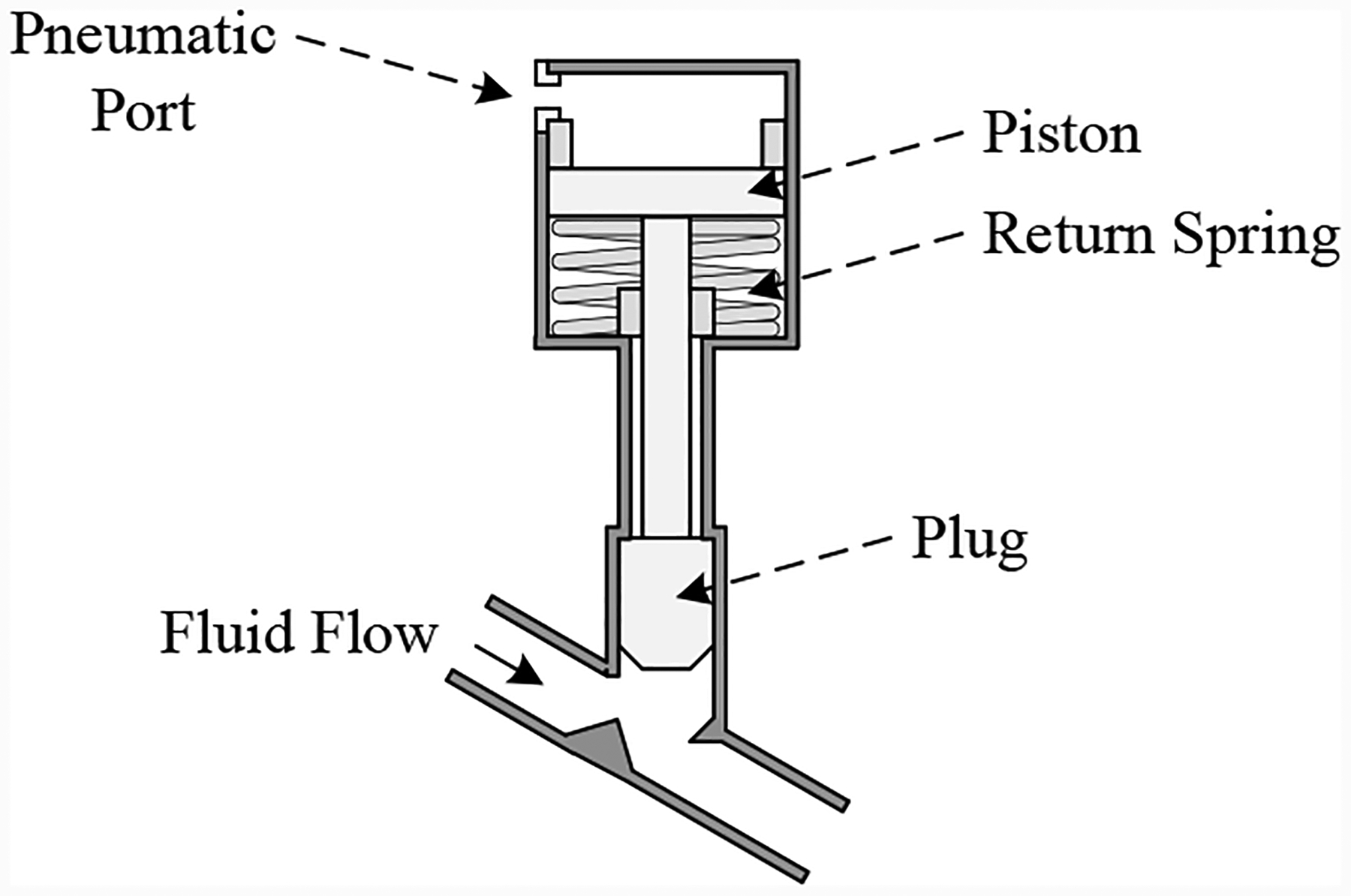
Discretely-controlled valve.

The discrete-controlled valve (DV), illustrated in Fig. 3 is a normally-open valve with a linear cylinder actuator. The valve is closed by filling the chamber above the piston with pressurized air up to the supply pressure, and opened by evacuating the chamber to atmosphere. The spring returns the valve to its default position.

**Figure 4.**
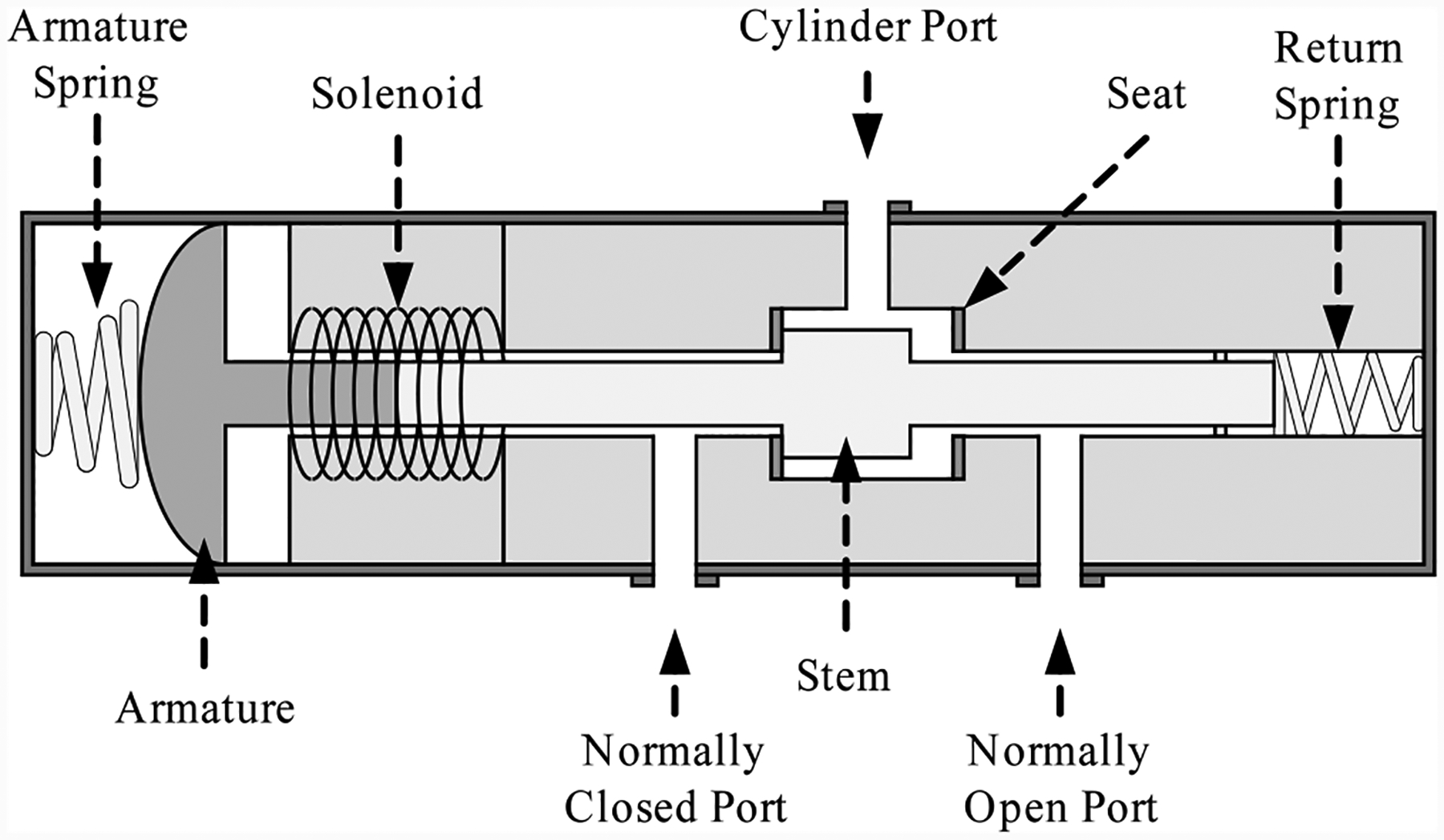
Three-way two-position solenoid valve.

A three-way two-position solenoid valve (SV), illustrated in Fig. 4, is used for controlling the operation of the DV valve. The cylinder port connects to the DV valve, the normally closed (NC) port connects to the supply pressure, and normally open (NO) port is left unconnected, allowing venting to atmosphere. When the solenoid is energized, the path from the NC port to cylinder port is open, allowing pressurized air to pass from the supply to the valve, thus actuating the valve. When de-energized, the supply pressure is closed off and the path from the cylinder port to the NO port is opened, thus venting the actuation pressure in the DV valve, allowing the valve to open due to the return spring. The solenoid is powered by 24 V DC either through the power supply or by a backup battery.

### 2.2. DV Fault Injection

Pneumatic valves can suffer from leaks, an increase in friction due to wear, and spring degradation (Daigle & Goebel, 2011). Because friction and spring faults cannot easily be injected or their rate of progression controlled, only to leak faults are discussed in this work. However, leaks are the most common faults found in pneumatic valves.

For the DV, two different leak faults may be considered: (*i*) a leak to atmosphere, and (*ii*) a leak from the supply. In the former, this can be manifested as a leak across the NO seat of the solenoid valve, or a leak in the pressure line going to the pneumatic valve. For the latter case, the fault can be manifested as a leak across the NC seat of the solenoid valve. To emulate these faults, two remotely-operated proportional valves, V1 and V2, were installed as shown in Fig. 1. One valve, V1, leaks to atmosphere (henceforth called the vent valve), while the other, V2, is installed on a bypass line around the solenoid valve (henceforth called the bypass valve). The position of the vent and bypass valves can be controlled through a current signal, continuously between 0 and 100% open. In this way, one can control the fault progression (growth of leak size) according to various progression profiles.

**Figure 5.**
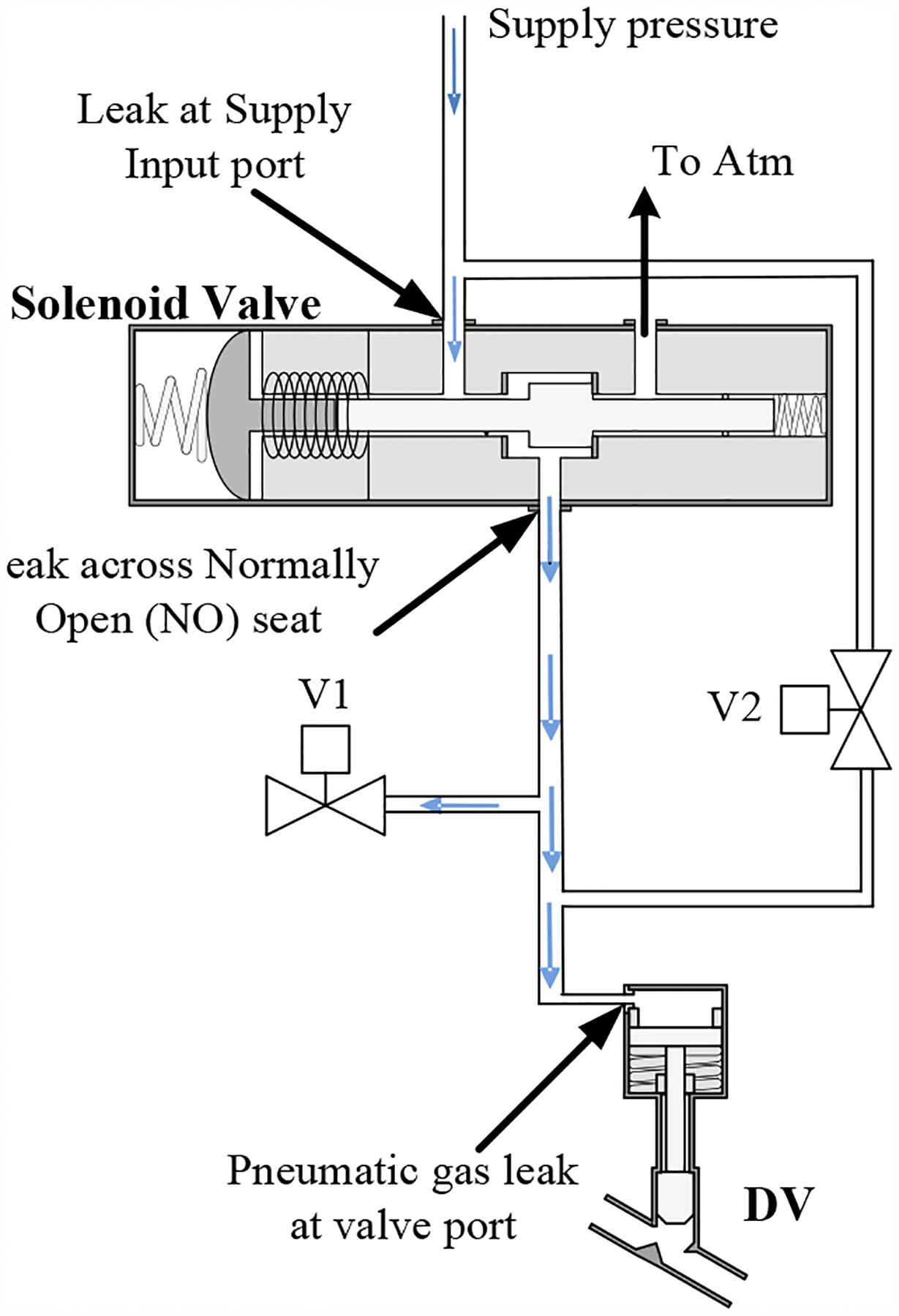
Leak to atmosphere fault on the DV injected through the vent valve V1.

Fig. 5 illustrates a leak to atmosphere using the vent valve (V1). The leak through V1 emulates a leak at the cylinder port or across the NO seat.

**Figure 6.**
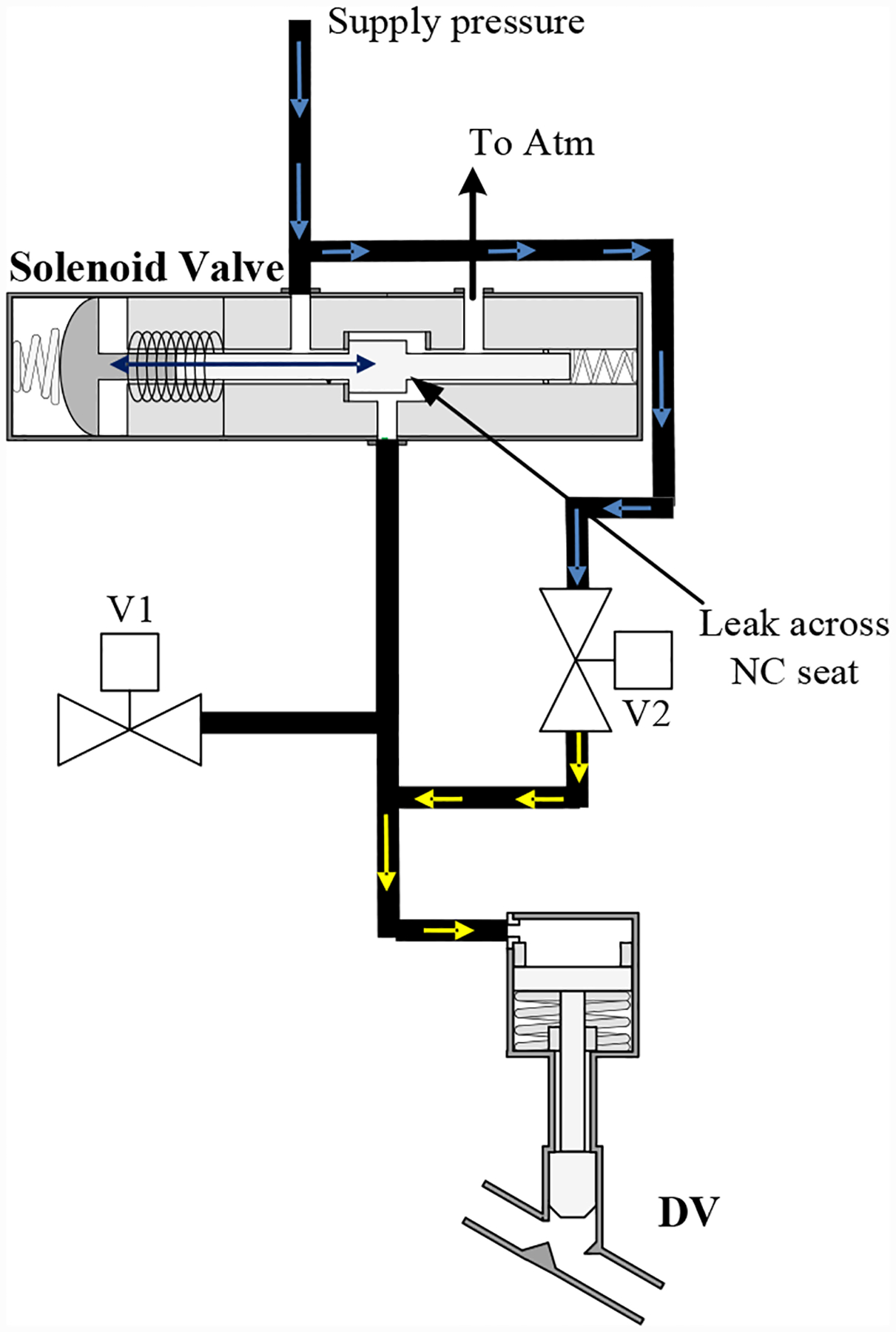
Solenoid valve leak fault injection when de-energized on DV valve.

Similarly, Fig. 6 illustrates a leak from the supply using the bypass valve (V2). The leak through V2 emulates a leak across the NC seat.

### 2.3. CV Operation

**Figure 7.**
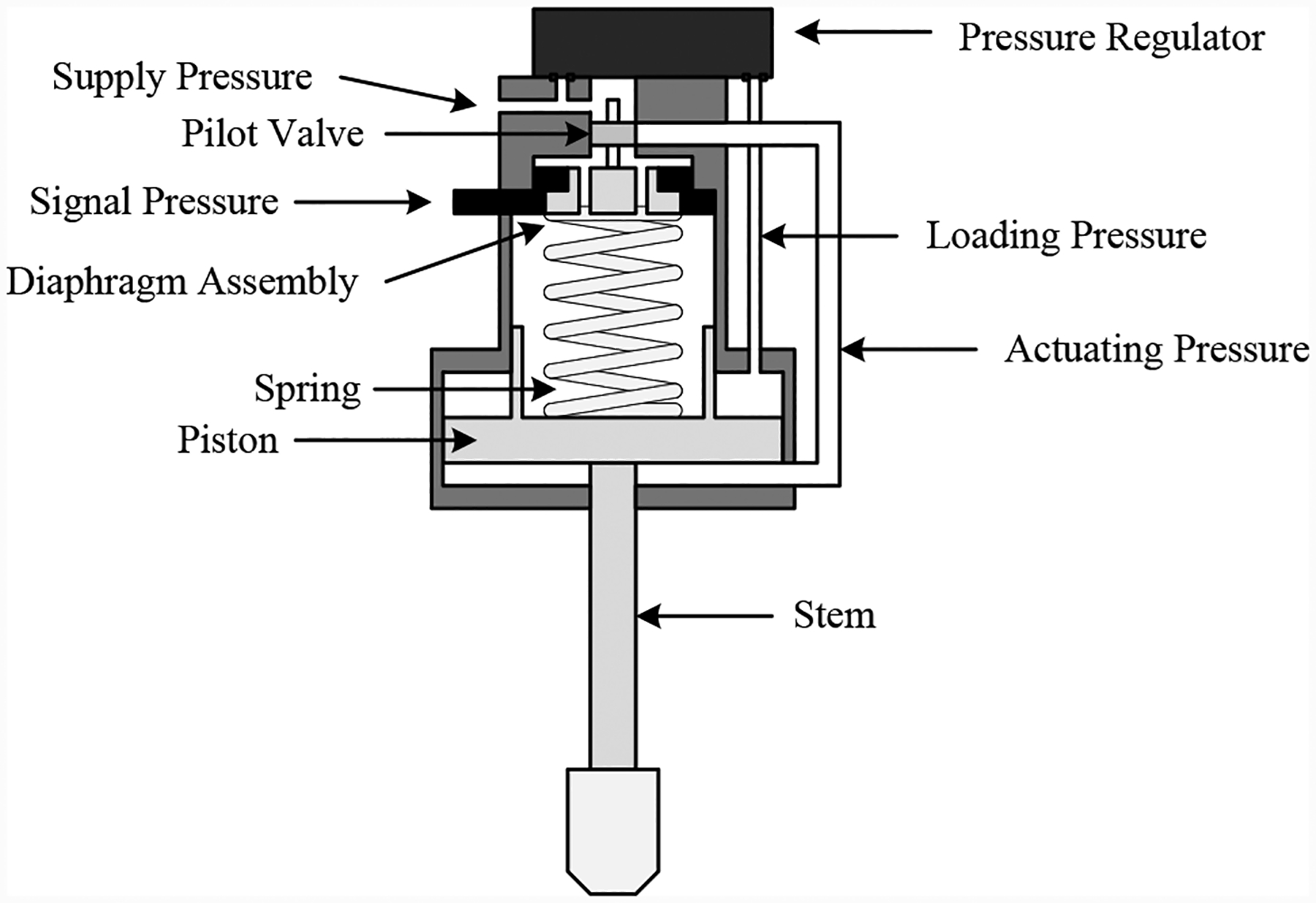
Continuously-controlled valve.

The CV, illustrated in Fig. 7, is a normally-closed valve with a linear cylinder actuator with dual pressure chambers. The valve is positioned by a pressure difference between the primary pressure chamber which is at standard operational pressure and the secondary chamber which can vary in pressure as controlled through the IPT.

The IPT output pressure is regulated down from the input pressure and is directly proportional to the applied control current supplied to the transducer. Thus, a low current will create a lower output pressure and a higher current will increase the output pressure.

### 2.4. CV Fault Injection

**Figure 8.**
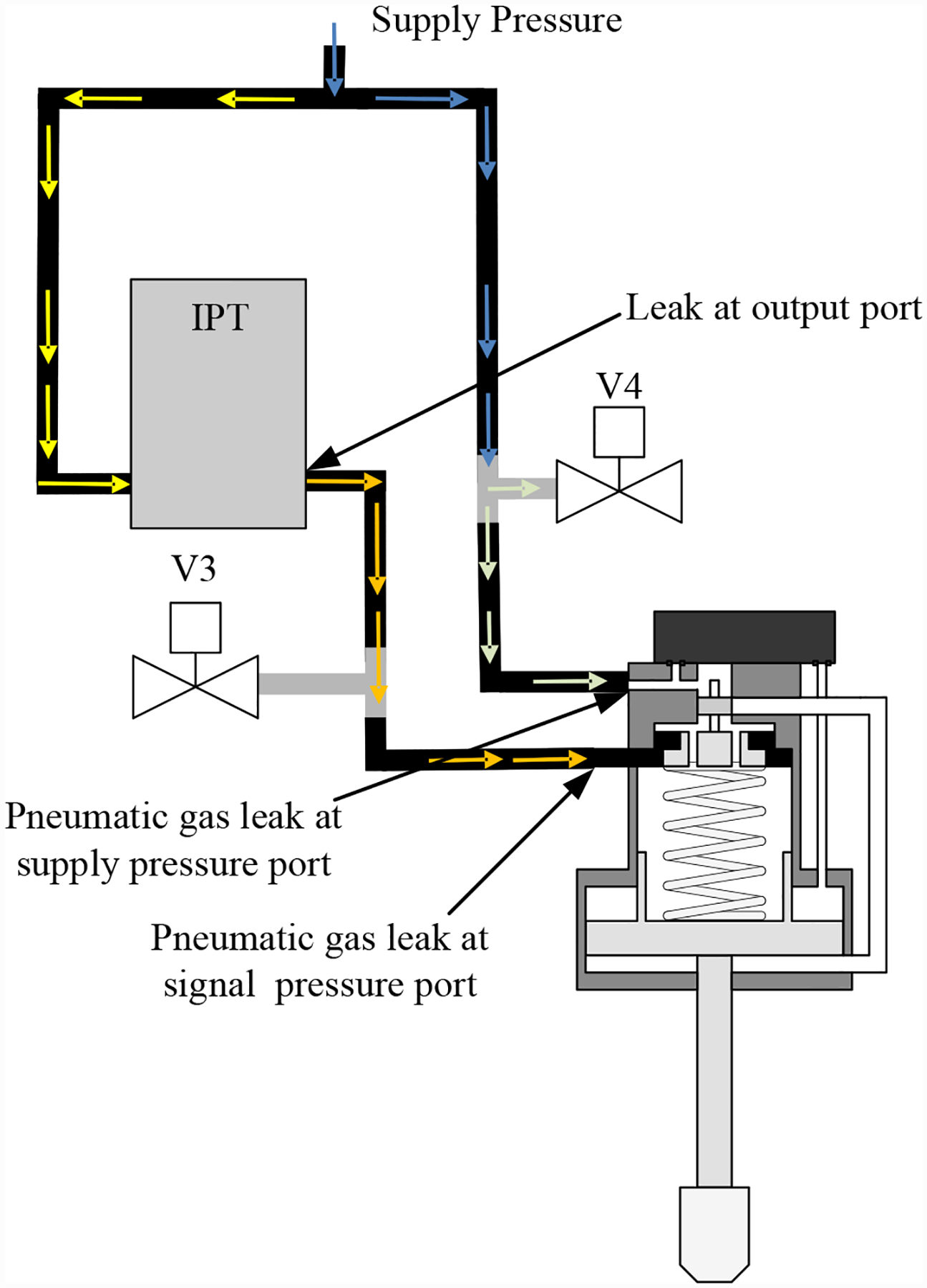
CV valve leaks.

As shown in Fig. 8, two different leak faults for the CV are considered: (*i*) a leak to atmosphere from the signal line, through valve V3; and (*ii*) a leak to atmosphere from the supply line, through valve V4. Like V1 and V2, V3 and V4 are proportional valves that can be controlled from 0 to 100% to implement any desired fault progression profile.

## 3. Valve Modeling

In this work, a model-based approach to valve prognostics (Daigle & Goebel, 2011) is developed and implemented, which requires dynamic models of the components that describe both nominal and degraded operation. A physics-based approach is adopted where the model is described using ordinary differential equations. For implementation purposes, discrete-time versions are converted using a sample time of 1 *×* 10^−4^ s.

Models for both discretely- and continuously-opening pneumatically actuated valves are developed, which were were originally presented in (Daigle et al., 2014; Kulkarni, Daigle, Gorospe, & Goebel, 2015), and are summarize here for completeness. Along with providing dynamics of the respective components, the section presents how EOL is defined for these valves.

In (Daigle & Goebel, 2011) the authors concluded that corrosion-based leaks are not a function of usage, i.e., cycling, but are correlated to environmental conditions only. Usage would have an effect on other fault modes which are out of scope for this study. The work discussed herein focuses on faults that can be controlled in experiments.

### 3.1. Discrete Valve Modeling

A normally-open discretely-opening valve (as seen in Fig. 3) is considered in this work. Normally, the chamber above the piston is open to atmosphere, and so the piston is forced up by the return spring. The valve is closed by filling the chamber up to the supply pressure. The pressure force overcomes the spring force, moving the piston downward, closing the valve. The valve is opened by evacuating the gas in the chamber to atmosphere.

The valve model is based on mass and energy balances. The system state includes the position of the valve, *x*(*t*), the velocity of the valve, *v*(*t*), the mass of the gas in the volume above the piston, and the mass of the gas in the pipe connecting the solenoid valve to the pneumatic valve port:

(1) $$x(t)=\left[\begin{array}{c} x(t)\\ v(t)\\ m_{t}(t)\\ m_{p}(t) \end{array}\right].$$

The position is defined as *x* = 0 when the valve is fully closed, and *x* = *L*_*s*_ when fully open, where *L*_*s*_ is the stroke length of the valve.

The derivatives of the states are described by

(2) $$\dot{x}(t)=\left[\begin{array}{c} v(t)\\ a(t)\\ f_{t}(t)\\ f_{p}(t) \end{array}\right],$$

where *a*(*t*) is the valve acceleration, *f*_*t*_(*t*) is the mass flow going into the pneumatic port from the pipe, and *f*_*p*_(*t*) is the total mass flow into the pipe.

The single input is considered to be

(3) $$u(t)=\left[u_{t}(t)\right],$$

where *u*_*t*_(*t*) is input pressure to the pneumatic port, which alternates between the supply pressure and atmospheric pressure depending on the commanded valve position.

The acceleration is defined by the combined mass of the piston and plug, *m*, and the sum of forces acting on the valve, which includes the force from the pneumatic gas, *F*_*p*_ = (*p*_*t*_(*t*)-*p*_*atm*_)*A*_*p*_, where *p*_*t*_(*t*) is the gas pressures on the top of the piston, and *A*_*p*_ is the surface area of the piston; the weight of the moving parts of the valve, *F*_*w*_ = −*mg*, where *g* is the acceleration due to gravity; the spring force, *F*_*s*_ = *k*(*x*(*t*)+*x*_*o*_), where *k* is the spring constant and *x*_*o*_ is the amount of spring compression when the valve is open; friction, *F*_*f*_ = −*rv*(*t*), where *r* is the coefficient of kinetic friction, and the contact forces *F*_*c*_(*t*) at the boundaries of the valve motion,

(4) $$F_{c}(t)=\{\begin{array}{ll} k_{c}(-x), & \text{ if }x<0,\\ 0, & \text{ if }0\leq x\leq L_{s},\\ -k_{c}\left(x-L_{s}\right), & \text{ if }x>L_{s}, \end{array}$$

where *k*_*c*_ is the (large) spring constant associated with the flexible seals. Overall, the acceleration term is defined by

(5) $$a(t)=\frac{1}{m}\left(F_{s}-F_{p}-F_{f}-F_{w}+F_{c}\right).$$

The pressure *p*_*t*_(*t*) and the pipe pressure, *p*_*p*_(*t*), are calculated as:

(6) $$p_{t}(t)=\frac{m_{t}(t)R_{g}T}{V_{t_{0}}+A_{p}\left(L_{s}-x(t)\right)}$$

(7) $$p_{p}(t)=\frac{m_{p}(t)R_{g}T}{V_{p}}$$

where an isothermal process is assumed in which the (ideal) gas temperature is constant at *T*, *R*_*g*_ is the gas constant for the pneumatic gas, $V_{t_{0}}$ is the minimum gas volume for the gas chamber above the piston, and *V*_*p*_ is the pipe volume.

The gas flows are given by:

(8) $$f_{p,in}(t)=f_{g}\left(u_{t}(t),p_{p}(t)\right)$$

(9) $$f_{p,\text{leak}}(t)=f_{g}\left(p_{p}(t),p_{\text{leak}}\right)$$

(10) $$f_{p,t}(t)=f_{g}\left(p_{p}(t),p_{t}(t)\right)$$

(11) $$f_{p}(t)=f_{p,in}(t)-f_{p,t}(t)-f_{p,\text{leak}}(t)$$

(12) $$f_{t}(t)=f_{p,t}(t)$$

where *f*_*p,in*_ is the flow into the pipe from the supply or atmosphere, *f*_*p,leak*_ is a leak term with *p*_*leak*_ being the pressure outside the leak, *f*_*p,t*_ is the flow from the pipe to the chamber above the piston, and *f*_*g*_ defines gas flow through an orifice for choked and non-choked flow conditions (Perry & Green, 2007). Non-choked flow for *p*_1_ ≥ *p*_2_ is given by

(13) $$f_{g,nc}(p_{1},p_{2})=C_{s}A_{s}p_{1}\sqrt{\frac{\gamma }{ZR_{g}T}\left(\frac{2}{\gamma -1}\right)\left({\left(\frac{p_{2}}{p_{1}}\right)}^{\frac{2}{\gamma }}-{\left(\frac{p_{2}}{p_{1}}\right)}^{\frac{\gamma +1}{\gamma }}\right)},$$

where γ is the ratio of specific heats, *Z* is the gas compressibility factor, *C*_*s*_ is the flow coefficient, and *A*_*s*_ is the orifice area. Choked flow for *p*_1_ ≥ *p*_2_ is given by

(14) $$f_{g,c}\left(p_{1},p_{2}\right)=C_{s}A_{s}p_{1}\sqrt{\frac{\gamma }{ZR_{g}T}{\left(\frac{2}{\gamma +1}\right)}^{\frac{\gamma +1}{\gamma -1}}}.$$

Choked flow occurs when the upstream to downstream pressure ratio exceeds ${\left(\frac{\gamma +1}{2}\right)}^{\gamma /(\gamma -1)}$. The overall gas flow equation is then given by

(15) $$f_{g}\left(p_{1},p_{2}\right)=\{\begin{array}{ll} f_{g,nc}\left(p_{1},p_{2}\right) & \text{if }p_{1}\geq p_{2}\\ \; & \text{and }\frac{p_{1}}{p_{2}}<{\left(\frac{\gamma +1}{2}\right)}^{\frac{\gamma }{\left(\gamma -1\right)}},\\ f_{g,c}\left(p_{1},p_{2}\right) & \text{if }p_{1}\geq p_{2}\\ \; & \text{and }\frac{p_{1}}{p_{2}}\geq {\left(\frac{\gamma +1}{2}\right)}^{\frac{\gamma }{\left(\gamma -1\right)}},\\ -f_{g,nc}\left(p_{2},p_{1}\right) & \text{if }p_{2}>p_{1}\\ \; & \text{and }\frac{p_{2}}{p_{1}}<{\left(\frac{\gamma +1}{2}\right)}^{\frac{\gamma }{\left(\gamma -1\right)}},\\ -f_{g,c}\left(p_{2},p_{1}\right) & \text{if }p_{2}>p_{1}\\ \; & \text{and }\frac{p_{2}}{p_{1}}\geq {\left(\frac{\gamma +1}{2}\right)}^{\frac{\gamma }{\left(\gamma -1\right)}}, \end{array}.$$

As shown by Eq. 13 and Eq. 14, the leak rate is determined by pressure differences, gas properties, and valve parameters *C*_*leak*_ and *A*_*leak*_. As the leak grows (by the corresponding leak valve opening), this is reflected as a change in *A*_*leak*_. Based on the developed testbed experimental data, it is observed that the leak area is proportional to the square of the valve position, i.e.,

(16) $$A_{\text{leak}}=K_{\text{leak}}x_{\text{leak}}^{2},$$

for some proportionality constant *K*_*leak*_. As the leak area increases it directly affects the position travelled by the valve during operation. The relationship in Eq. 16 is specific to the valves under test. A generalized relationship is discussed in (Richer & Hurmuzlu, 1999)

The only available measurement is the valve position, given by

(17) y(t)=[x(t)].

**Figure 9.**
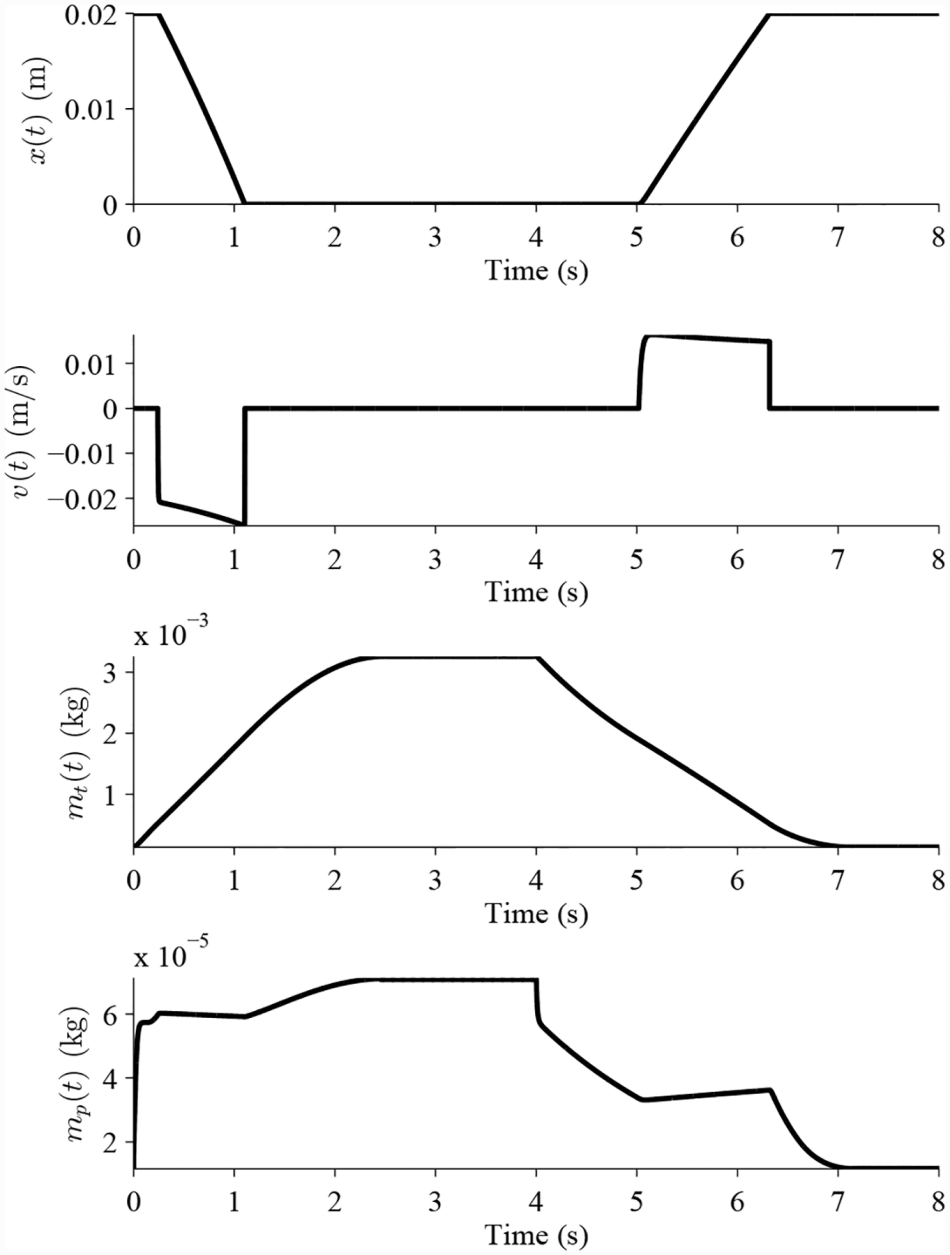
Nominal DV operation.

Fig. 9 shows an example nominal valve cycle. The valve starts in its default open state. The valve is commanded to close at 0 s. Supply pressure (75 psig) is delivered to the pipe and to the valve, causing the piston to lower, closing the valve just after 1 s. At 4 s, the valve is commanded to open, and the pipe is opened to atmosphere. The pipe pressure and valve pressure drop, and once the pressure drops low enough, the spring overcomes the pressure force and the piston moves upwards. The valve completes opening just after 6 s. The valve parameters were identified from known valve specifications, and unknown parameters estimated to match the nominal opening and closing times, which for the actual valve, are both around 3.5 s.

**Figure 10.**
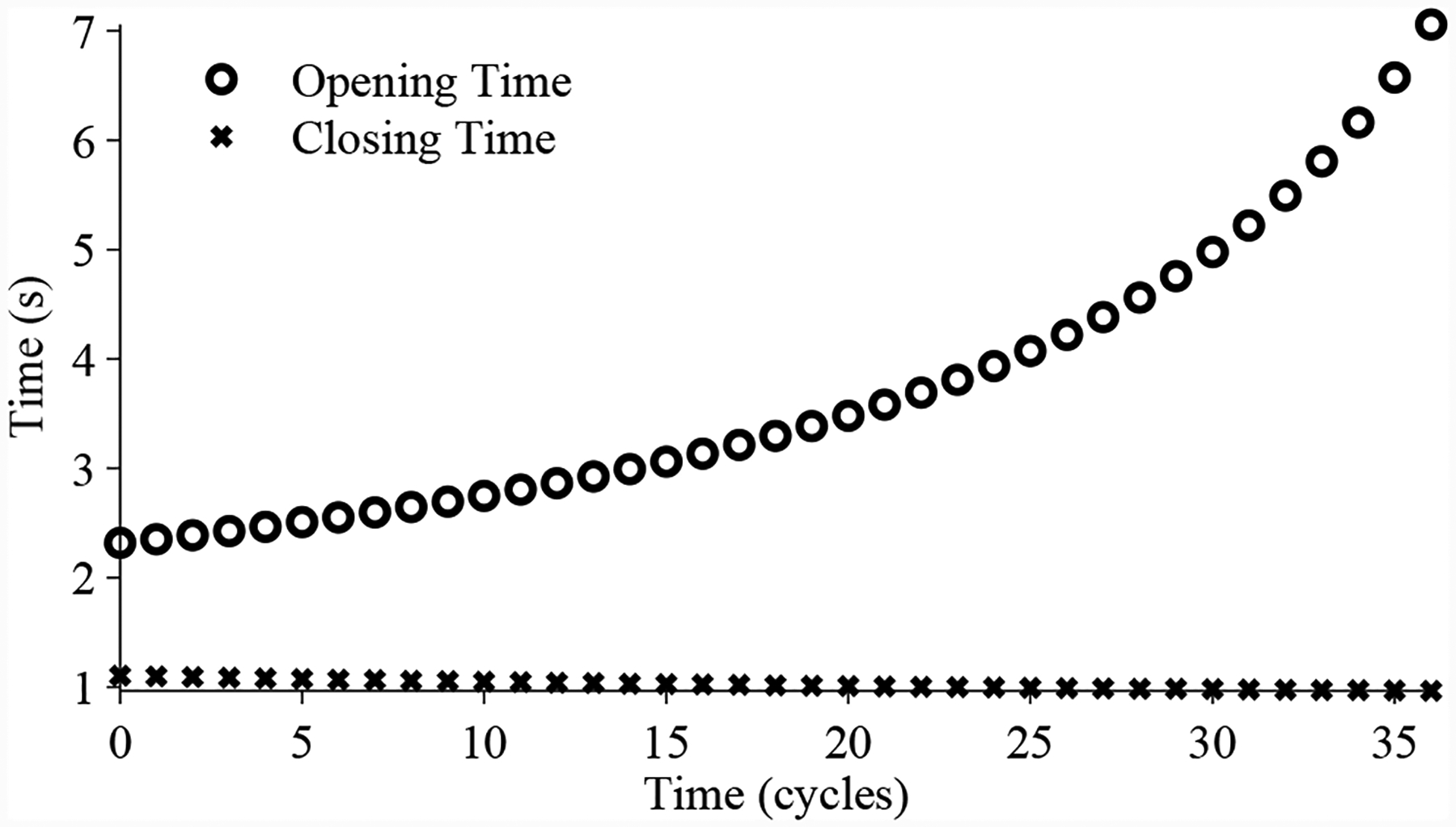
DV timing with leak from supply, with linearly increasing leak coefficient.

**Figure 11.**
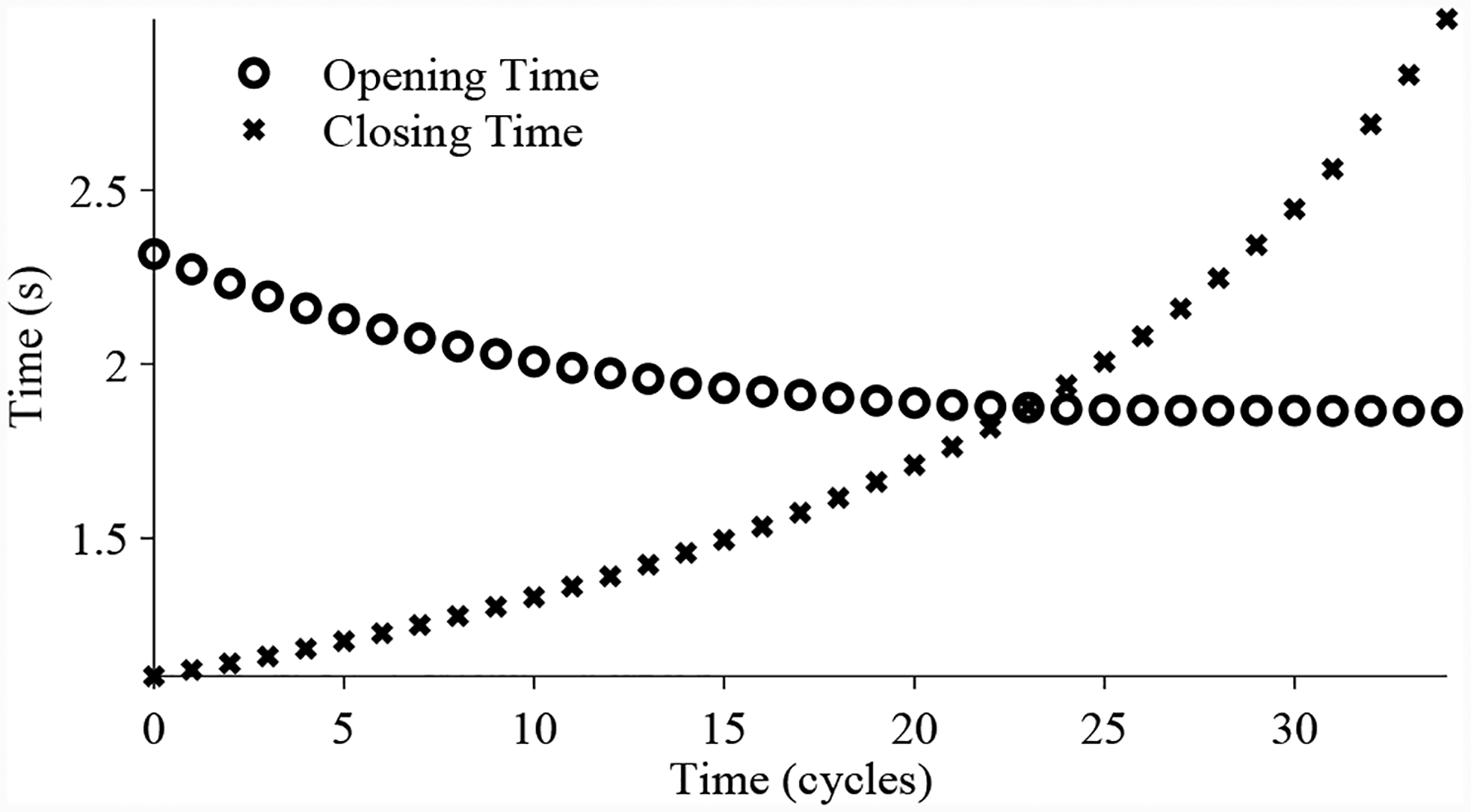
DV timing with leak to atmosphere, with linearly increasing leak coefficient.

As discussed in Section 2, two different leak faults are considered, one in which there is a leak from the supply pressure input to the valve (*p*_*leak*_ is the supply pressure), emulated using the bypass valve, and one in which there is a leak out to atmosphere (*p*_*leak*_ is atmospheric pressure), emulated using the vent valve. In the former case, the valve will close more slowly and open faster, and in the latter, the valve will open more slowly and close faster. With a large enough leak, the valve may fail to open or close completely. Fig. 10 shows the changes in valve timing with the leak from the supply, and Fig. 11 shows the changes in valve timing with the leak to atmosphere.

In this work a damage progression model is considered where the leak hole area increases linearly with time (Fontana, 1986; Ahammed, 1998). The growth curve used in this work is completely based on assumed operating corrosion conditions like humidity, salt in air, temperature which stay more or less constant over the experiment cycles. Any fluctuations like seasonal effects are averaged out because the degradation phenomena is progressing at a very slow rate and does not change with each operating cycle. This growth curve can be controlled systematically through the developed tested by injecting specific profile of damage progression. With additional knowledge the damage progression provided based on corrosion type similar profile can be programmed into the system.

End of life (EOL) is defined through open/close time limits of the valves, as in real valve operations (Daigle & Goebel, 2011). The valve in the testbed is required to open within 8.5 s and close within 6 s.

### 3.2. Continuous Valve Modeling

The actuator has two pressure ports, one for the supply pressure, and one for the signal pressure as seen in Fig. 7 for a normally-closed continuously-controlled valve. External to the valve, the signal pressure is controlled between 3–15 psig in order to move the valve between fully closed and fully open. A pressure regulator maintains a loading pressure on top of the valve piston, and the piston moves by modulating the actuating pressure via the pilot valve. The pilot valve, balanced by the spring and the diaphragm assembly, moves up or down according to the signal pressure. When moving up, the volume below the piston is opened up the atmosphere, and when the pilot moves down, the volume below the piston is opened up to the supply pressure.

Similar to the DV, the CV model is based on mass and energy balances. The system state includes the position of the piston, *x*_*p*_(*t*), velocity of the piston, *v*_*p*_(*t*), position of the pilot/spring assembly, *x*_*s*_(*t*), velocity of the pilot/spring assembly, *v*_*s*_(*t*), mass of gas in volume below the piston *m*_*b*_(*t*), mass of gas in the pipe connecting to the supply input, *m*_*sp*_(*t*), and mass of gas in the pipe connecting to the signal input, *m*_*sg*_(*t*):

(18) $$x(t)=\left[\begin{array}{c} x_{p}(t)\\ v_{p}(t)\\ x_{s}(t)\\ v_{s}(t)\\ m_{b}(t)\\ m_{sp}(t)\\ m_{sg}(t) \end{array}\right].$$

The piston position is defined as *x*_*p*_ = 0 when the valve is fully closed, and *x*_*p*_ = *L*_*s*_ when fully open, where *L*_*s*_ is the stroke length of the valve (about 20 mm). When fully closed, the pilot/spring assembly position is also defined as *x*_*s*_ = 0.

The derivatives of the states are described by

(19) $$\dot{x}(t)=\left[\begin{array}{c} v_{p}(t)\\ a_{p}(t)\\ v_{s}(t)\\ a_{s}(t)\\ f_{b}(t)\\ f_{sp}(t)\\ f_{sg}(t) \end{array}\right],$$

where *a* is acceleration and *f* is mass flow.

The two inputs are considered to be

(20) $$u(t)=\left[\begin{array}{l} u_{sp}(t)\\ u_{sg}(t) \end{array}\right],$$

where *u*_*sp*_(*t*) is input pressure to the supply port, which is nominally 75 psig, and *u*_*sg*_(*t*) is the input pressure to the signal port, which varies between 3–15 psig, depending on the commanded valve position.

The acceleration of the piston is defined by the combined mass of the piston and plug, *m*_*p*_, and the sum of forces acting on the piston, which includes the force from the actuating pressure, *F*_*a*_ = *p*_*b*_*A*_*p*_, where *A*_*p*_ is the area of the piston in contact with the actuating pressure; the force from the loading pressure, *F*_*l*_ = *A*_*l*_*p*_*l*_, where *A*_*l*_ is the area of the piston in contact with the loading pressure; friction, *F*_*f*_ = −*r*_*p*_*v*_*p*_(*t*), where *r*_*p*_ is the coefficient of kinetic friction; the spring force, *F*_*s*_ = *k*(*x*_*p*_ + *x*_*o*_ − *x*_*s*_) where *x*_*o*_ is the spring compression at the closed position; the weight, *F*_*w*_ = −*m*_*p*_*g*, and the contact forces, *F*_*c*_(*t*), at the boundaries of the valve/piston motion,

(21) $$F_{c}(t)=\{\begin{array}{ll} k_{c}(-x), & \text{ if }x<0,\\ 0, & \text{ if }0\leq x\leq L_{s},\\ -k_{c}\left(x-L_{s}\right), & \text{ if }x>L_{s}, \end{array}$$

where *k*_*c*_ is the (large) spring constant associated with the flexible seals. Overall, the acceleration term is defined by

(22) $$a_{p}(t)=\frac{1}{m_{p}}\left(F_{a}-F_{s}-F_{l}-F_{w}-F_{f}+F_{c}\right)$$

The pressures *p*_*l*_ is assumed to be constant and known, and the pressure *p*_*b*_ is computed as

(23) $$p_{b}=\frac{m_{b}(t)R_{g}T}{V_{b_{0}}+A_{p}x_{p}(t)},$$

where an isothermal process is assumed in which the (ideal) gas temperature is constant at *T*, *R*_*g*_ is the gas constant for the pneumatic gas, and $V_{t_{0}}$ is the minimum gas volume for the gas chamber below the piston.

The acceleration of the pilot/spring assembly is defined by their combined mass, *m*_*s*_, and the sum of forces acting on the assembly, which includes the force from the spring *F*_*s*_ (as defined above); the force from the signal pressure, *F*_*sg*_ = (*p*_*sg*_ − *p*_*atm*_)*A*_*d*_, where *A*_*d*_ is the area of the diaphragm in contact with the signal pressure and *p*_*atm*_ is atmospheric pressure; friction, *F*_*fs*_ = *r*_*s*_*v*_*s*_(*t*), where *r*_*s*_ is the coefficient of kinetic friction; the force from the supply pressure, *F*_*sp*_ = (*p*_*sp*_ − *p*_*atm*_)*A*_*sp*_, where *A*_*sp*_ is the area of the pilot in contact with the supply pressure; the weight, *F*_*ws*_ = *m*_*s*_*g*; and contact forces *F*_*cs*_ (defined as above but with *L*_*ss*_, the stroke length of the pilot/spring assembly).

The pressures *p*_*sg*_ and *p*_*sp*_ are computed as

(24) $$p_{sg}=\frac{m_{sg}(t)R_{g}T}{V_{sg}},$$

(25) $$p_{sp}=\frac{m_{sp}(t)R_{g}T}{V_{sp}},$$

where *V*_*sg*_ is the volume of the pipe containing the signal pressure, and *V*_*sp*_ is the volume of the pipe containing the supply pressure.

The mass flows *f*_*b*_(*t*), *f*_*sp*_(*t*), and *f*_*sg*_(*t*) are defined by

(26) $$f_{b}(t)=\left(x_{s}<0\right)\cdot f_{g}\left(p_{sp}(t),p_{b}(t)\right)-\left(x_{s}>0\right)\cdot f_{g}\left(p_{b}(t),p_{atm}\right),$$

(27) $$f_{sp}(t)=f_{g}\left(u_{sp}(t),p_{sp}(t)\right)-f_{sp,\text{leak}}(t)-\left(x_{s}<0\right)\cdot f_{g}\left(p_{sp}(t),p_{b}(t)\right),$$

(28) $$f_{sg}(t)=f_{g}\left(u_{sg}(t),p_{sg}(t)\right)-f_{sg,\text{leak}}(t),$$

where *f*_*sg,leak*_ and *f*_*sg,leak*_ are leak terms (both leaks to atmosphere). Note also that the flows into and out of the underside of the piston depend on the position of the pilot/spring assembly. Here, *f*_*g*_ defines gas flow through an orifice for choked and non-choked flow conditions (Eq. 15).

The only available measurement is the valve position, given by

(29) $$y(t)=\left[x_{p}(t)\right].$$

**Figure 12.**
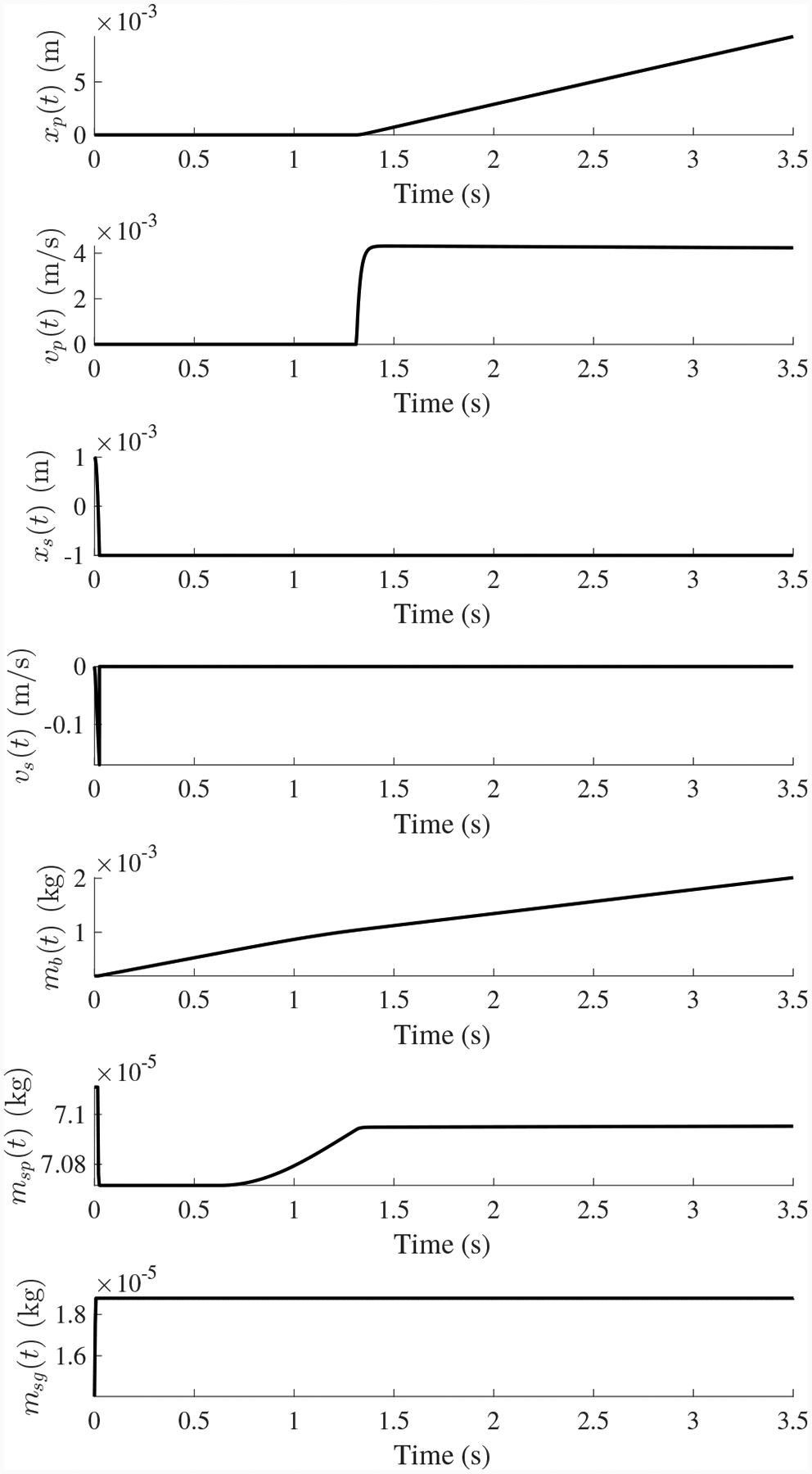
Nominal CV operation.

Fig. 12 shows an example nominal valve cycle. The valve starts in its default closed state. The valve is commanded to 50% open using a signal pressure of 9 psig. The pilot valve moves, allowing gas from the supply line to enter below the piston, increasing the mass of gas below the piston and increasing the pressure. When there is enough pressure, the piston begins to move up, and when the valve reaches 50% open, the forces balance and the pilot valve closes. Due to small fluctuations in pressure the pilot intermittently moves up and down to keep the pressures balanced, causing sllight disturbances in position.

**Figure 13.**
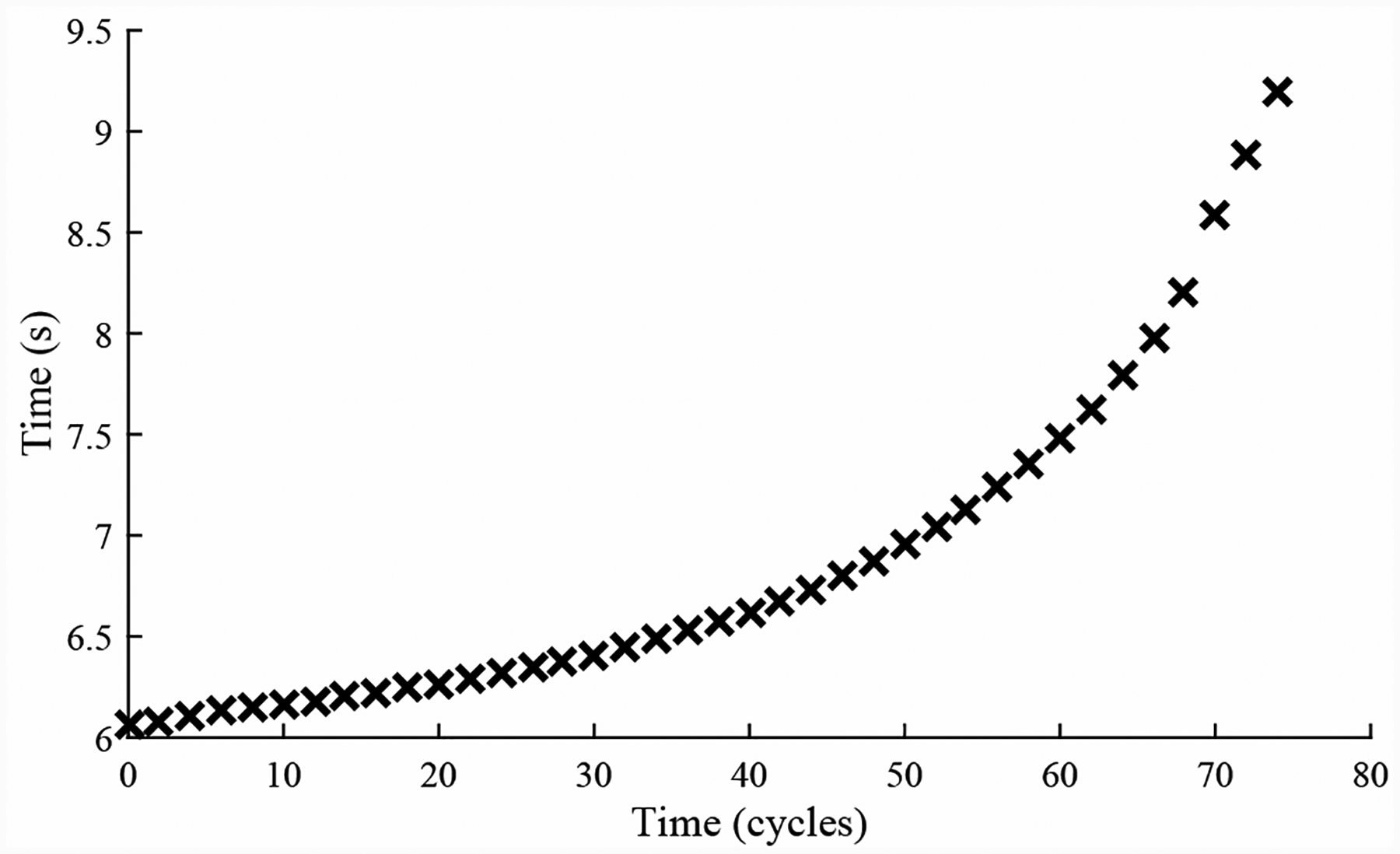
CV open times with a progressing leak from the supply line.

**Figure 14.**
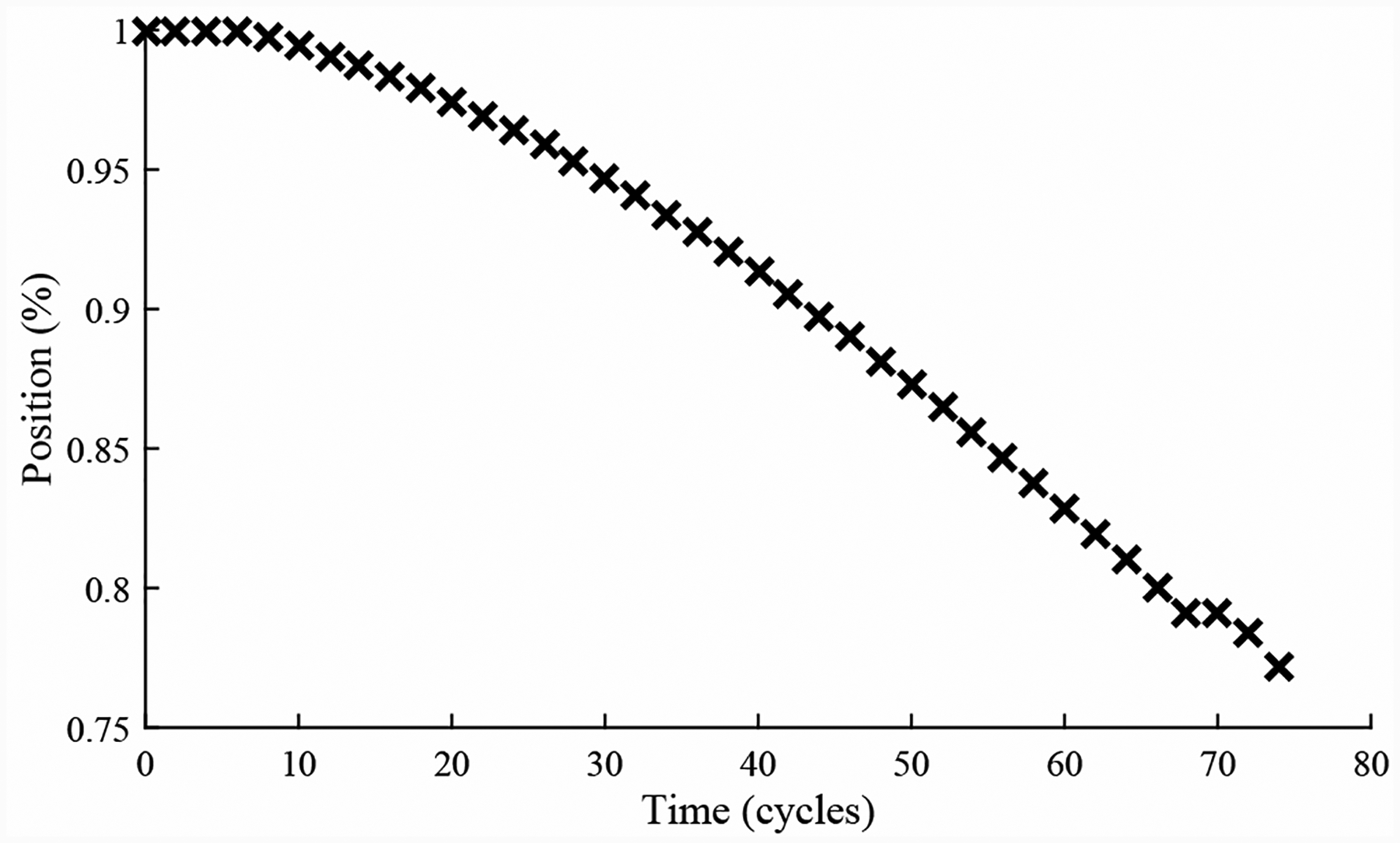
CV steady-state position with a progressing leak from the supply line.

Leak faults will cause an effect on the behavior of the valve. With a leak from the supply line, trends observed are seen in Figs. 13 and 14. Due to the decrease in effective supply pressure, it takes longer to close the valve and the steady-state position decreases because the valve is set up based on a nominal supply pressure. With a leak from the signal line, the effect on valve timing is not very significant, but since signal pressure will be lower due to the leak, its steady-state position will decrease.

End of life (EOL) is defined through the use of timing limits on the valves, as is done in real valve operations (Daigle & Goebel, 2011), and also the error in its steady-state position. The valve in the testbed is required to open within 7.5 s, close within 5 s, and when commanded to open to 100% it must open up at least to 98.5%.

## 4. Valve Prognosis

In this section the prognosis framework developed for the valves, following the general estimation-prediction framework of model-based prognostics as defined in the scientific literature (Luo, Pattipati, Qiao, & Chigusa, 2008; Orchard & Vachtsevanos, 2009; Daigle & Goebel, 2013). However, since only valve timing values are used for prognosis, a simpler estimation approach (Daigle et al., 2014), similar to that developed in (Teubert & Daigle, 2013) is implemented, as opposed to more complex and computationally intensive filtering approaches used in previous works (Daigle, Saha, & Goebel, 2012; Orchard & Vachtsevanos, 2009). Section 4.1 formulates the prognostics problem, followed by a description of the estimation approach and a description of the prediction approach.

### 4.1. Problem Formulation

The system model assumed may be generally defined as

(30) x(k+1)=f(k,x(k),θ(k),u(k),v(k)),

(31) y(k)=h(k,x(k),θ(k),u(k),n(k)),

where *k* is the discrete time variable, $x(k)\in {\mathbb{R}}^{n_{x}}$ is the state vector, $\theta (k)\in {\mathbb{R}}^{n_{\theta }}$ is the unknown parameter vector, $u(k)\in {\mathbb{R}}^{n_{u}}$ is the input vector, $v(k)\in {\mathbb{R}}^{n_{v}}$ is the process noise vector, f is the state equation, $y(k)\in {\mathbb{R}}^{n_{y}}$ is the output vector, $n(k)\in {\mathbb{R}}^{n_{n}}$ is the measurement noise vector, and h is the output equation.^1^

In prognostics, the key factor is in predicting the occurrence of some event *E* that is defined with respect to the states, parameters, and inputs of the system. The event is defined as the earliest instant that some event threshold $T_{E}:{\mathbb{R}}^{n_{x}}\times {\mathbb{R}}^{n_{\theta }}\times {\mathbb{R}}^{n_{u}}\to B$, where B≜{0,1} changes from the value 0 to 1 (Daigle & Sankararaman, 2013). That is, the time of the event *k*_*E*_ at some time of prediction *k*_*P*_ is defined as

(32) $$k_{E}\left(k_{P}\right)≜\text{inf}\left\{k\in \mathbb{N}:k\geq k_{P}\wedge T_{E}(x(k),\theta (k),u(k))=1\right\}.$$

The time remaining until that event, Δ*k*_*E*_, is defined as

(33) $$\Delta k_{E}\left(k_{P}\right)≜k_{E}\left(k_{P}\right)-k_{P}.$$

In the context of systems health management, *T*_*E*_ is defined via a set of performance constraints that define what the acceptable states of the system are, based on x(*k*), *θ*(*k*), and u(*k*) (Daigle & Goebel, 2013). In this context, *k*_*E*_ represents end of life (EOL), and Δ*k*_*E*_ represents remaining useful life (RUL). As described in Section 3, for the valves, timing and steady-state position requirements define *T*_*EOL*_.

The prognostics problem is to compute estimates of EOL and/or RUL. This is done is two steps, an estimation step that computes estimates of x(*k*) and *θ*(*k*), followed by a prediction step that computes EOL/RUL using these values as initial states. For the case of the valve, the future inputs are known, i.e., the valve is simply cycled open and closed, so there is no uncertainty with respect to future inputs.

### 4.2. Fault Detection

Since valve position is measured, only valve timing values and steady-state position values are useful for prognostics. Timing information is obtained from the continuous position measurement data by extracting and computing the difference in time between when the valve is commanded to move, and when it reaches its final position. As discussed in Section 3.1, open and close times are used for faults in the DV, and, as discussed in Section 3.2, close times and steady-state position are used for faults in the CV.

To detect faults, predefined threshold are set on the opening times, closing times, and steady-state position. If the mean value, averaged over the last 3 cycles, is over the threshold, then a fault is detected.

### 4.3. Estimation

Using the model, measurements from valve timing and steady-state position are mapped back to the fault size (i.e., equivalent leak area). In order to perform the estimation, an offline lookup table is constructed using the simulation models of the valves to compute, for different values of leak size in the expected ranges, the open and close times (for the DV) and close times and steady-state position (for the CV) (Teubert & Daigle, 2013; Daigle et al., 2014). With a fine enough granularity, a lookup table will provide accurate estimates but at a fraction of the computational cost of online estimation methods.

The developed testbed allows for modular use of different corrosion propagation models. If a alternative corrosion growth is deemed to be a more desirable choice, it can be swapped in easily through replacement of a function call in the governing program. The prognostics approach is similarly flexible, because the open/close times are mapped to leak sizes. While it is assumed here that the leak sizes grow linearly, different leakage behavior can be used without impacting the rest of the prognostics framework

The calculated equivalent leak area is mapped back to the position of the leak valve. According to Eq. 16, the leak area increases linearly with the square of the leak valve position, hence square root of the leak size is calculated, i.e., $x_{\text{leak}}=\sqrt{A_{\text{leak}}/K_{\text{leak}}}$. The leak valve position, *x*_*leak*_, is assumed to be increasing linearly, so as to estimate the linear coefficients (where the slope is lumped with $\sqrt{K_{\text{leak}}}$. Given the estimated values of damage progression, a regression step is performed to find the line that fits this data, using the last *N* cycles.

For the leak to atmosphere of the DV, only closing times can be used (Daigle et al., 2014). This is because, in the presence of this leak, the valve may not get up to the full supply pressure when the valve closes in time for the next cycle, so since the internal valve actuator pressure is not measured, a correct initial condition is not available for the simulation with which to estimate the leak parameter value for the following opening time. For the supply leak of the DV, analogous situation arises and can use only opening times for leak parameter estimation.

For the signal line leak fault of the CV, steady state values are used. The signal pressure controls the open/close position of the valve while the supply pressure is used for regulating the pressure inside the valve. When this fault is injected, there is no change in the supply pressure but the signal pressure decreases and so the valve is not able to reach its desired steady state final value.

For the supply line leak fault of the CV, open time values are used. When this leak is injected, there is a decrease in the supply pressure, which leads to an increase in the valve opening time (since the corresponding pressure forces take longer to develop). As the leak increases the open time increases accordingly, while the steady state values remain relatively constant.

### 4.4. Fault Isolation

Faults are isolated by inspecting open/close timing and steady-state position trends (see Fig. 11, Fig. 10, Fig. 13, and Fig. 14). For the DV, since the two faults produce different qualitative changes on the valve timing, the observed trends tell us which fault is actually present.

For the CV, both faults have the same qualitative effects; they produce an increase in valve opening time and a decrease in steady-state position. However, their quantitative effects are different; the signal pressure leak has a greater effect on steady-state position and the supply pressure leak a greater effect on opening time. Therefore, based on the more significant trend faults can be isolated. For a signal leak, the deviation in nominal behavior will be observed first in steady-state position, and for a supply leak, the deviation will be observed first in the opening time. Depending upon the fault isolated the predictions for RUL are computed.

### 4.5. Prediction

Given the current estimated leak parameter value, and the regression parameters, leak parameter value at any future time can be calculated, using the damage progression equation (i.e., linearly progressing leak valve position). Using the lookup table, maximum valve open/close times and/or steady-state position values to maximum leak parameter values for the leak faults are mapped, and this defines the EOL thresholds in the leak parameter space. Using the relationship between leak size and leak valve position, obtain corresponding maximum values, and then solve for the time at which that threshold is crossed, given the fitted line, and thus compute EOL.

Prediction is not performed until a fault is detected. The regression is performed only over the data obtained since fault detection, so that nominal valve behavior is not used to estimate the fault progression parameters. The use of a filter on the data for fault detection introduces a slight lag, however in practice fault progression is very slow so this lag is negligible relative to the true EOL. In general, more robust fault detection strategies may also be used, but for our purposes a simple threshold works well.

## 5. Experimental Results

In this section, experimental results using the valve prognostics testbed are discussed. The valve is continually cycled open and closed in each experiment, with one cycle every 10 seconds, until the end of life condition is reached. For fault injection, the leak valve is opened at an increment of 1% at each cycle. The time 10 seconds is chosen such that the value has sufficient time to perform given operations under normal operating conditions. In the following sections results for the discrete valve and the continuous valve are presented respectively.

To evaluate the experiments, two metrics, prognostics horizon and relative accuracy (Saxena, Celaya, Saha, Saha, & Goebel, 2010) are computed. Relative accuracy is computed as the difference in the true and predicted values divided by the true value (in this case, for EOL):

(34) $$RA=\frac{\left|k_{E}^{*}-k_{E}\right|}{k_{E}^{*}},$$

where $k_{E}^{*}$ denotes the true value. We define prognostics horizon, *k*_*PH*_, as the first time point after fault detection (*k*_*d*_) in which the relative accuracy remains within a fraction *α* of the true value, in this case *α* = 0.15 is used. To compare experiments with different detection times and EOLs, the metric is normalized by computing it as the fraction:

(35) $$PH=\frac{\left|k_{PH}-k_{d}\right|}{k_{E}-k_{d}},$$

where a smaller value, which means accurate results earlier, is better. An averaged relative accuracy is computed over all prediction points from *k*_*d*_ to *k*_*E*_.

### 5.1. Discrete Valve

For faults in the discrete valve leak to atmosphere and leak to supply faults are discussed.

#### 5.1.1. Leak to Atmosphere

**Figure 15.**
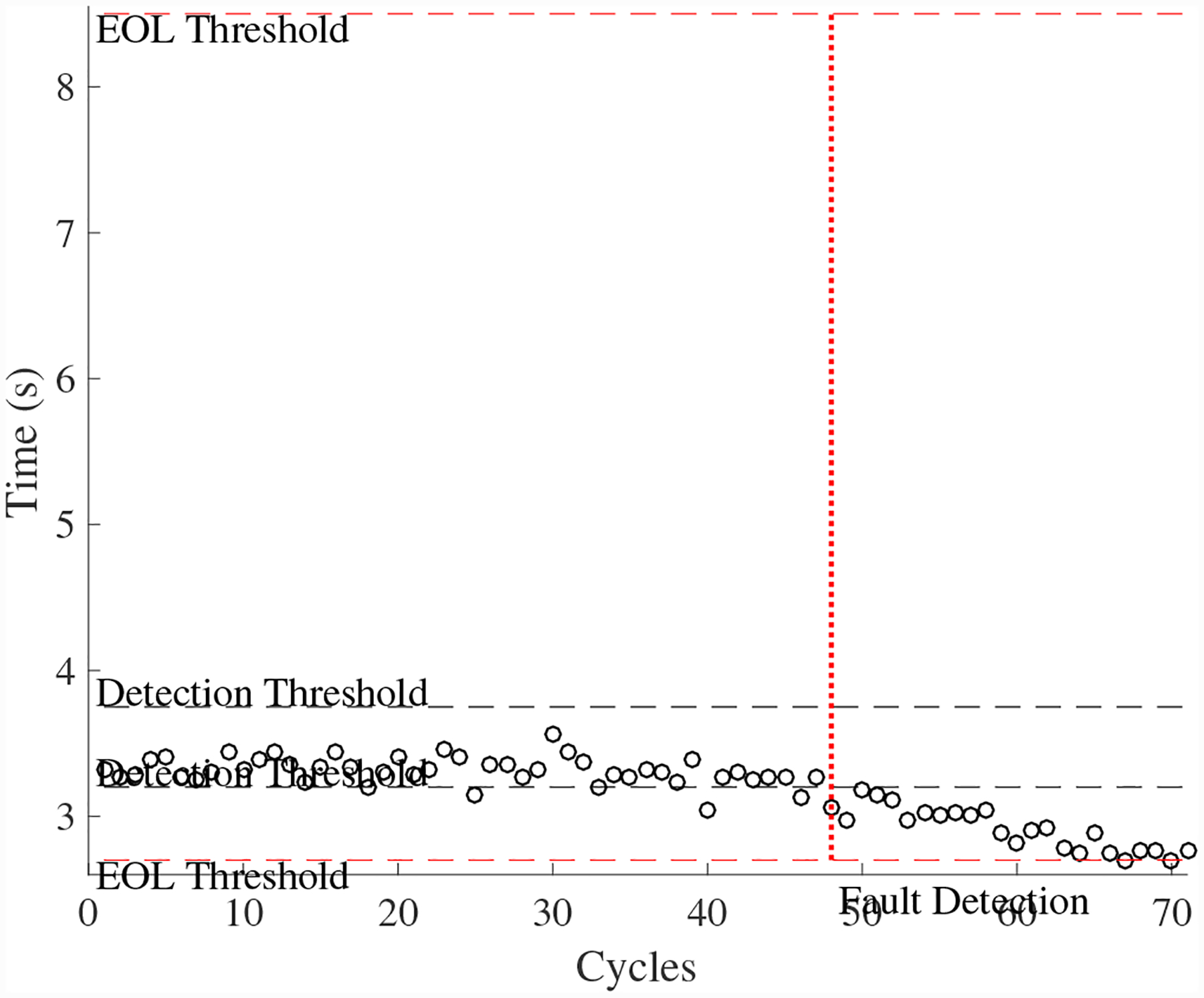
DV open times with a leak to atmosphere.

**Figure 16.**
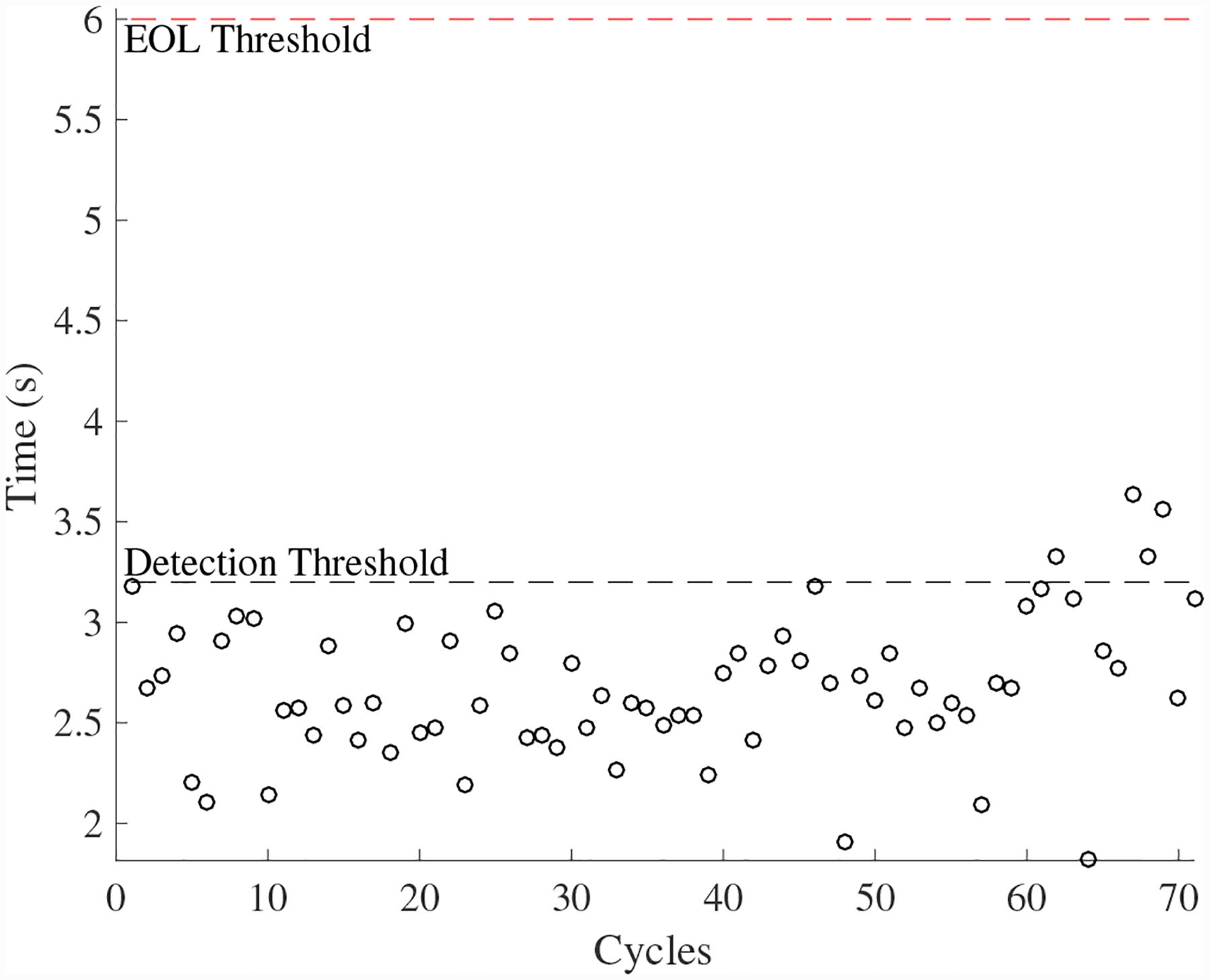
DV close times with a leak to atmosphere.

A total of 5 experiments were performed for this fault. As described in Section 2, the leak to atmosphere fault is injected by controlling the position of the leak valve V1. This emulates a leak across the NO seat of the solenoid valve. As described in Section 3, this fault causes an increase in closing times and a decrease in opening times. Fig. 15 shows the open times of the valve during the fault progression, with a noticable downwardward progression, in agreement with the model. Fig. 16 shows the close times, but any trend is masked by the noise in the computed closing times. A fault is detected at the 59th cycle based on the opening times.

**Figure 17.**
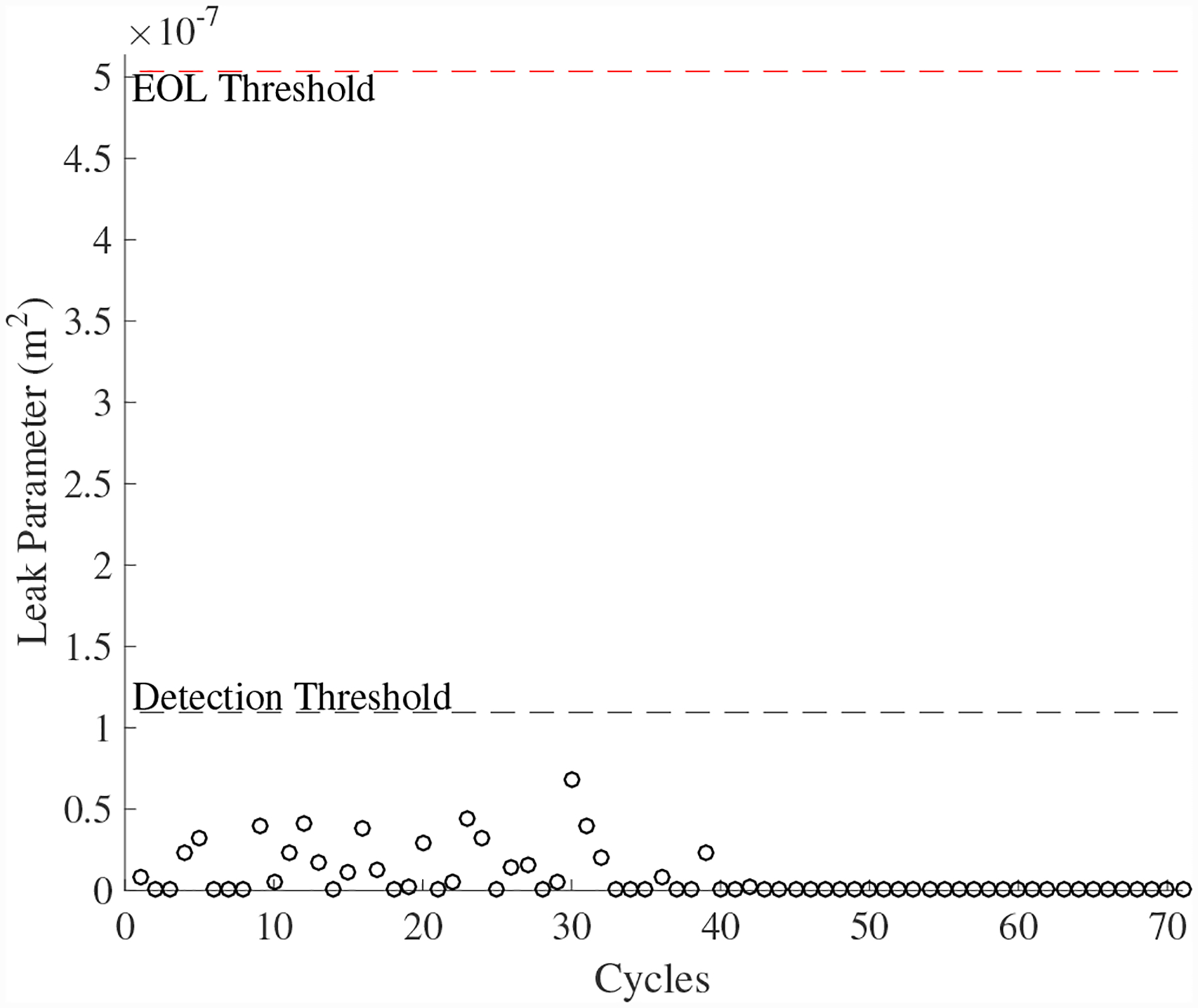
Estimated leak parameter values based on valve opening times for the leak to atmosphere for the DV.

**Figure 18.**
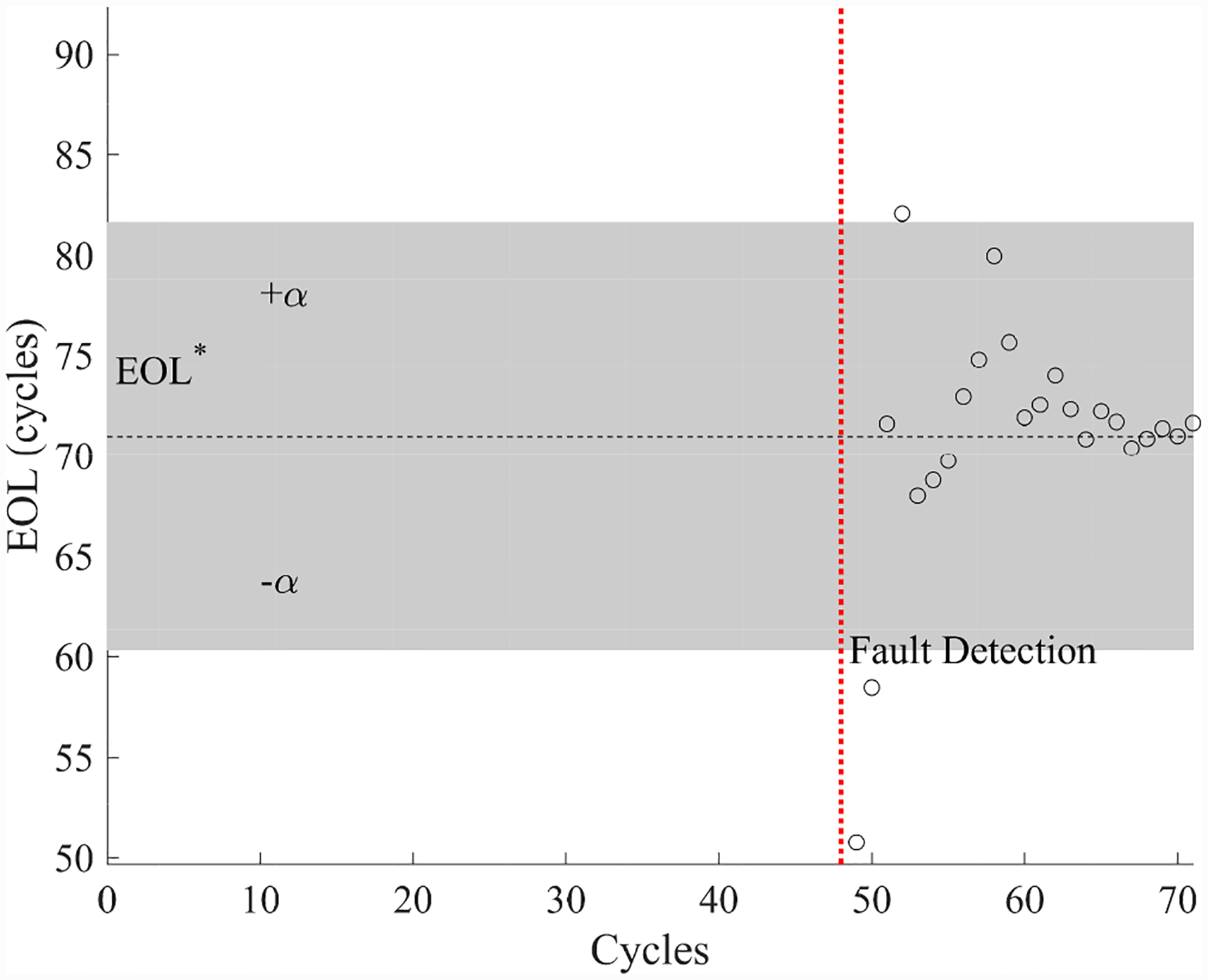
Predicted EOL values for the leak to atmosphere for the DV.

**Figure 19.**
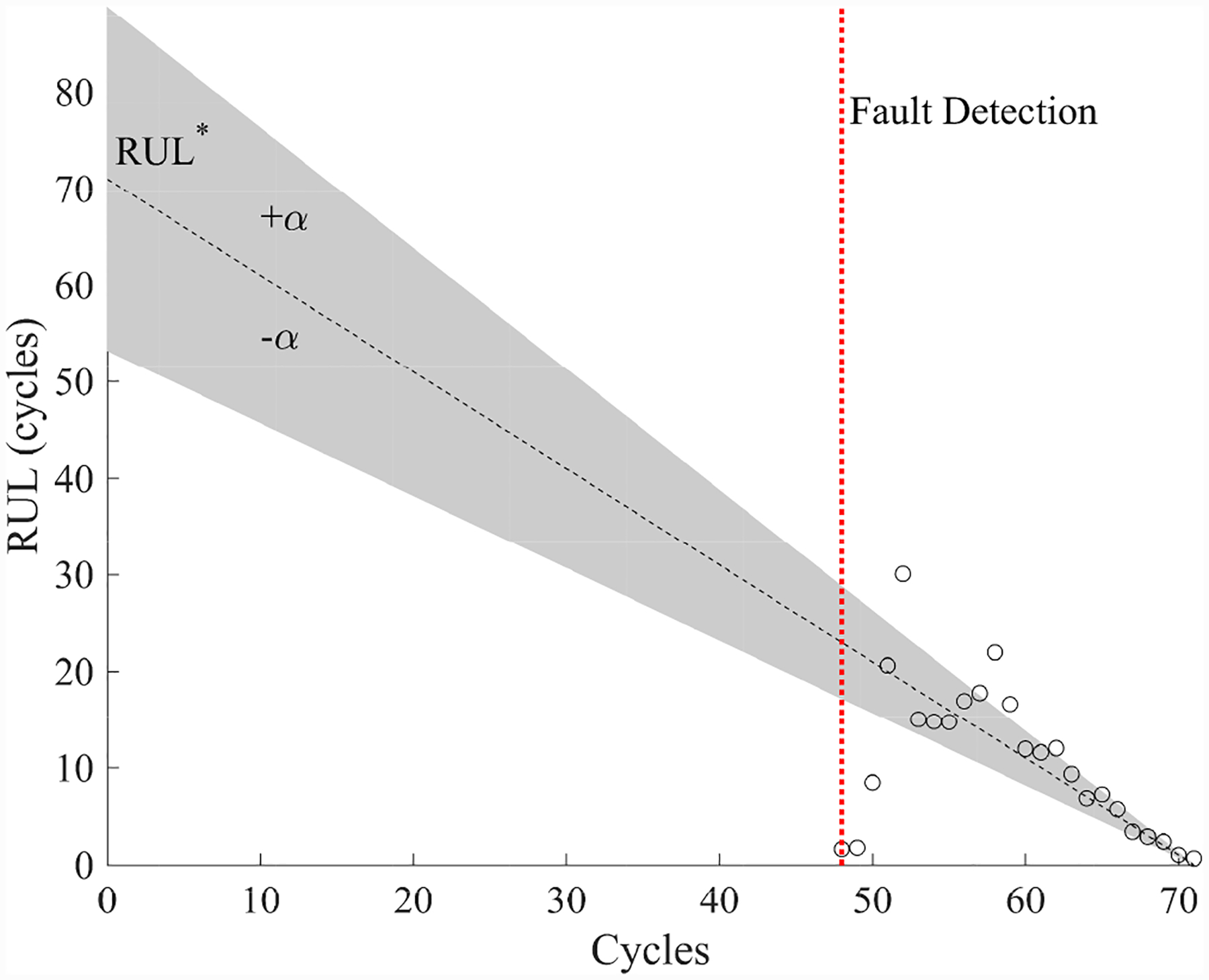
Predicted RUL values for the leak to atmosphere for the DV.

The estimated leak parameter values, based on the open times of the DV, are shown in Fig. 17. In order to estimate the fault progression parameters, the all values since detection are used. The EOL predictions are given in Fig. 18 and the RUL values in Fig. 19, where *α* = 0.15 represents a desired accuracy constraint, EOL* denotes the true EOL, and RUL* denotes the true RUL. The predictions converge soon after the fault is detected, with *PH* = 47.83%. RA averages to 98.33%.

Over all experiments, PH averages to 66.31% and average RA to 95.42%. For this fault, the progression of the fault is not very large relative to the nominal opening times, and so predictions are accurate only after halfway to EOL.

#### 5.1.2. Leak from Supply

**Figure 20.**
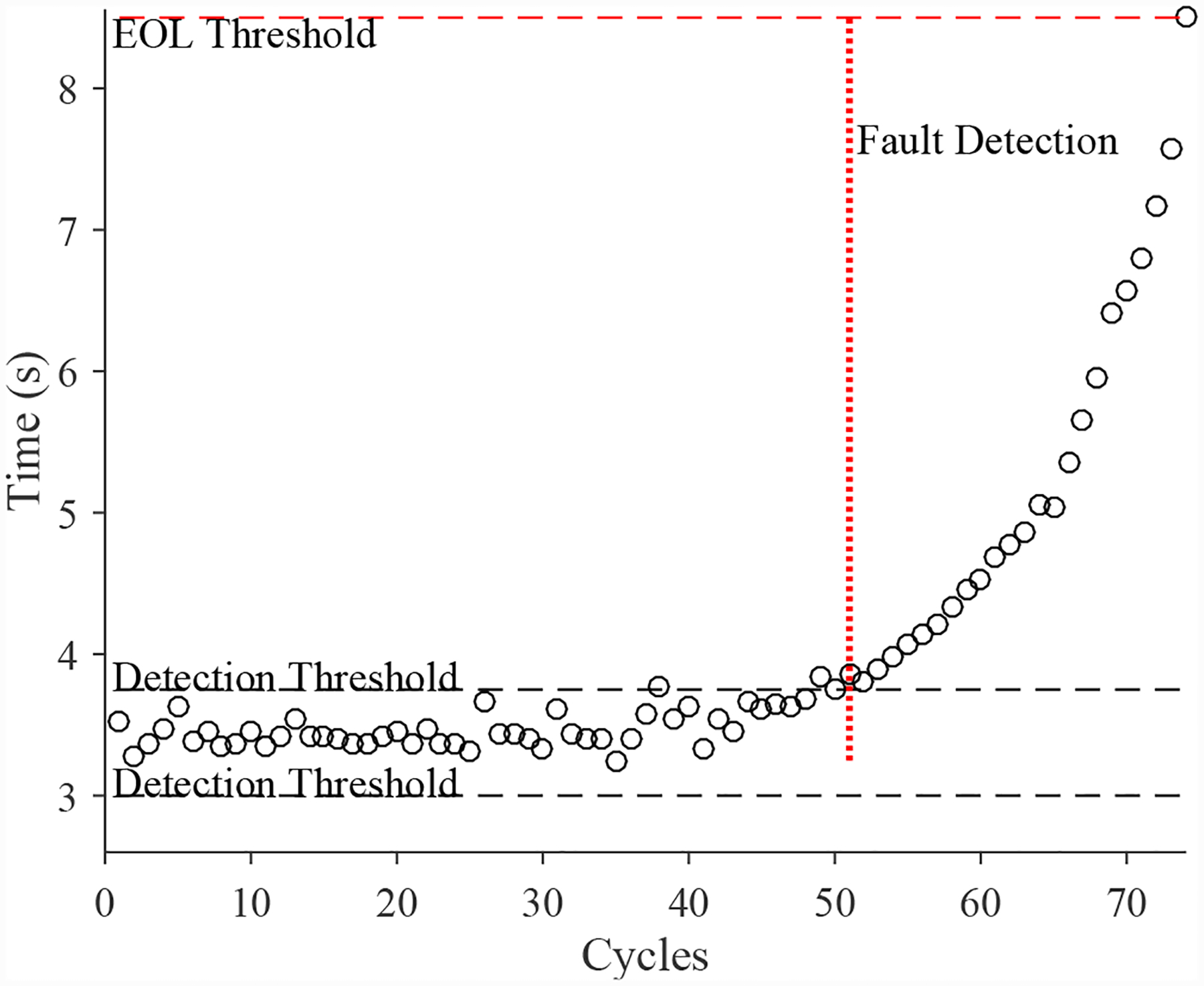
Valve open times with a leak from supply.

**Figure 21.**
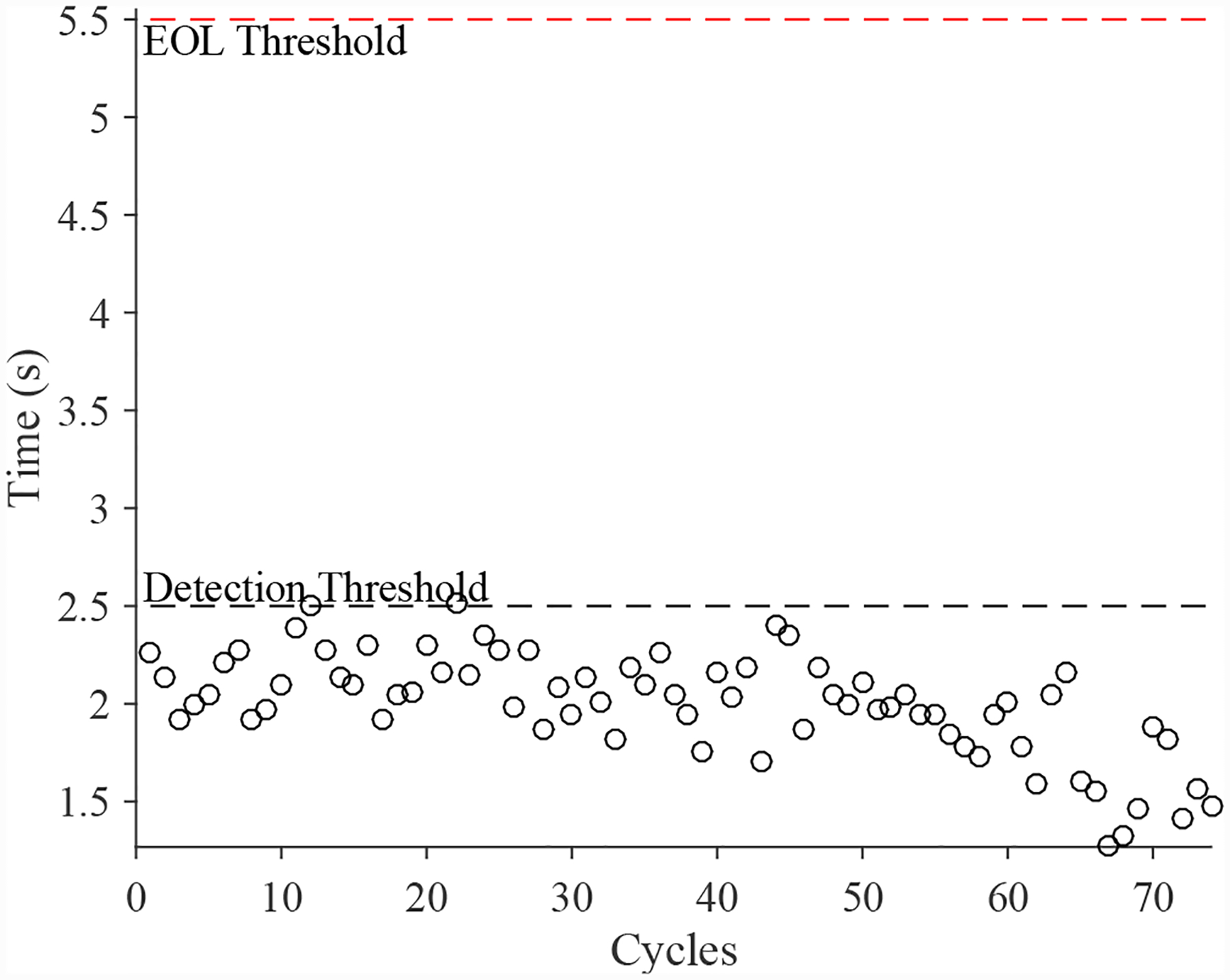
Valve close times with a leak from supply.

A total of 6 experiments were performed for this fault. As described in Section 2, the leak from supply fault is injected by controlling the position of the leak valve V2. This emulates a leak across the NC seat of the solenoid valve. As described in Section 3, this fault causes an increase in opening times and a slight decrease in closing times. Fig. 20 shows the open times of the valve during the fault progression, with a clear upward progression, in agreement with the model. Fig. 21 shows the close times, but any trend is masked by the noise in the computed closing times. A fault is detected at the 52nd cycle based on the opening times.

**Figure 22.**
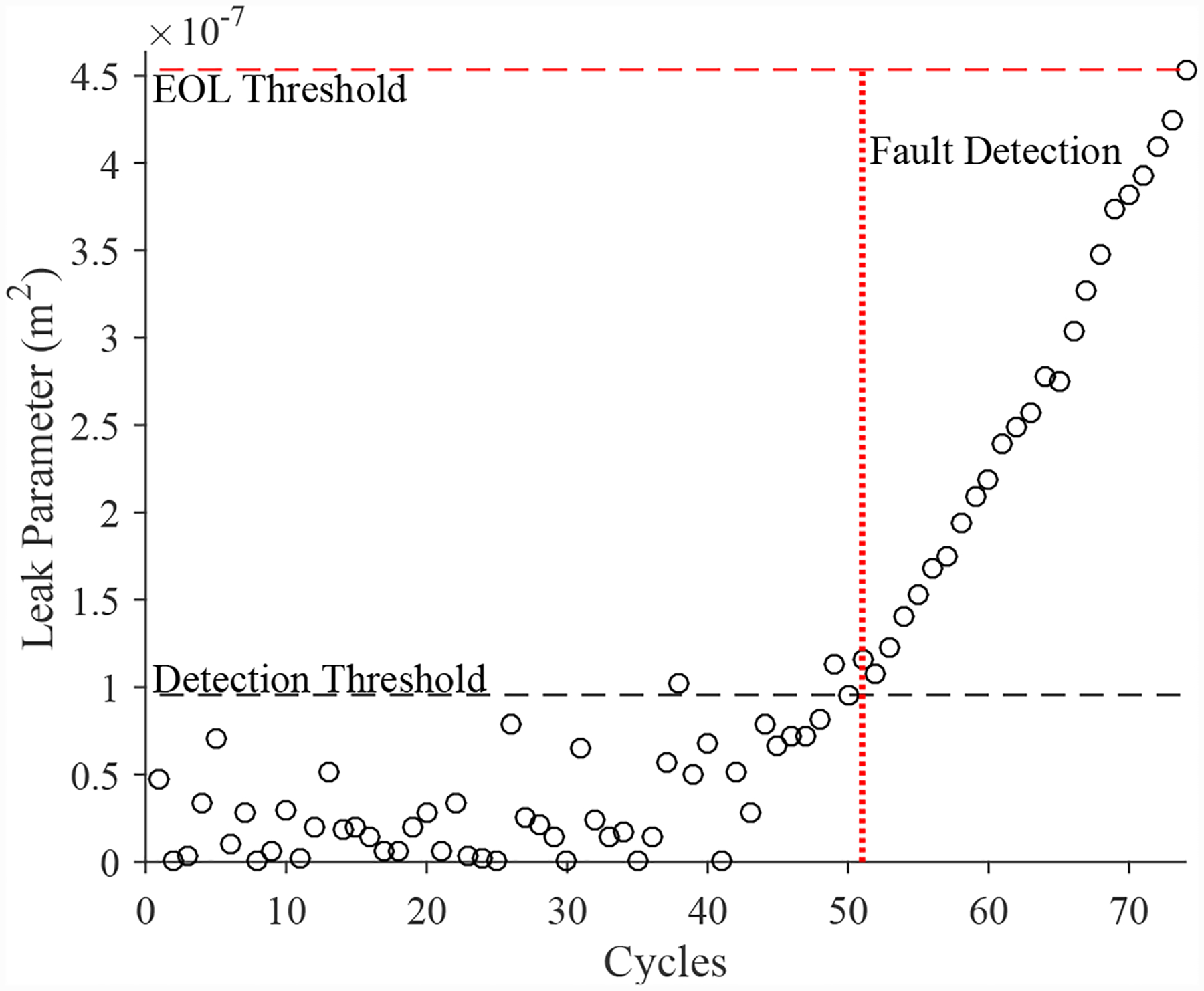
Estimated leak parameter values based on valve opening times for the leak from supply for the DV.

**Figure 23.**
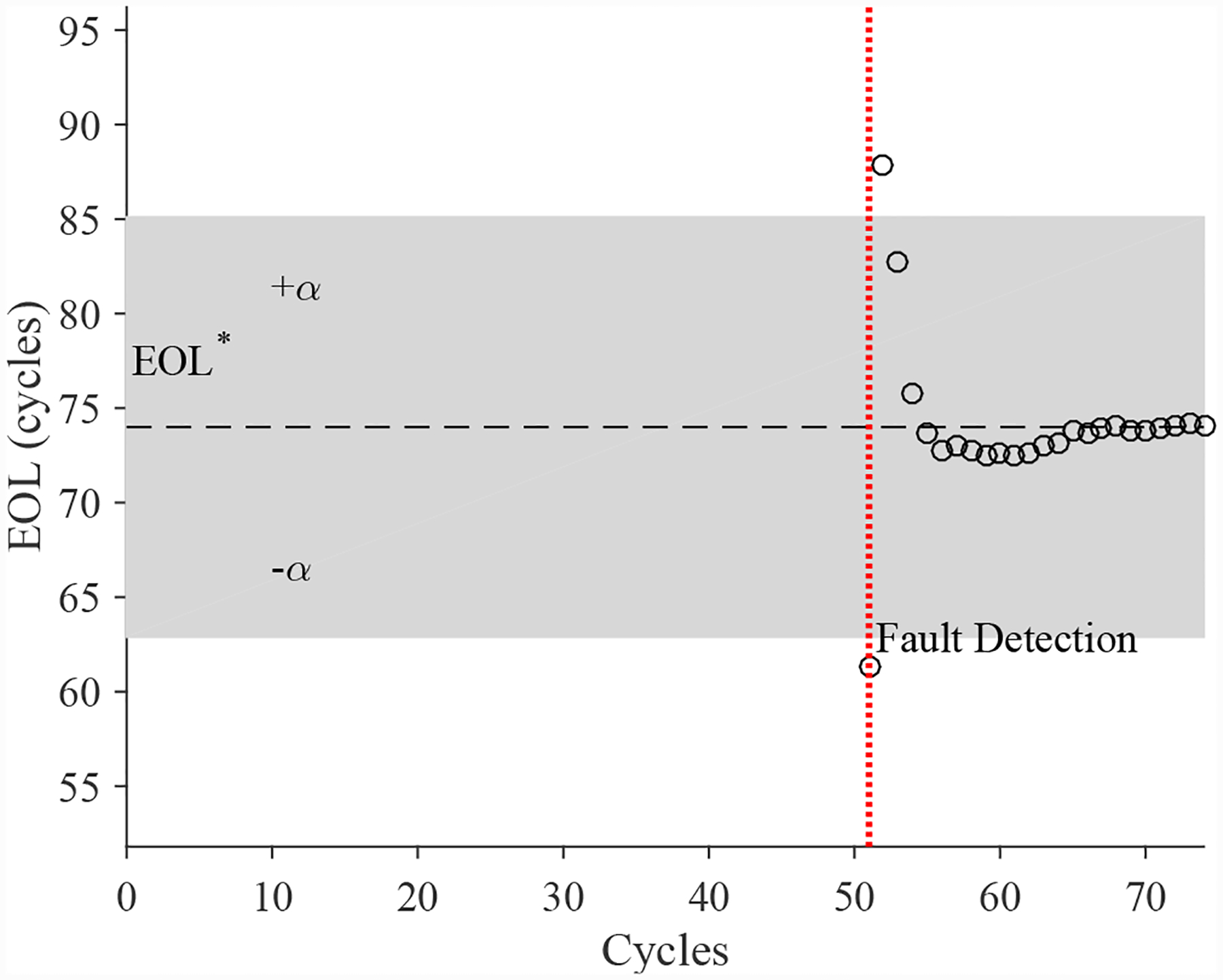
Predicted EOL values for the leak from supply for the DV.

**Figure 24.**
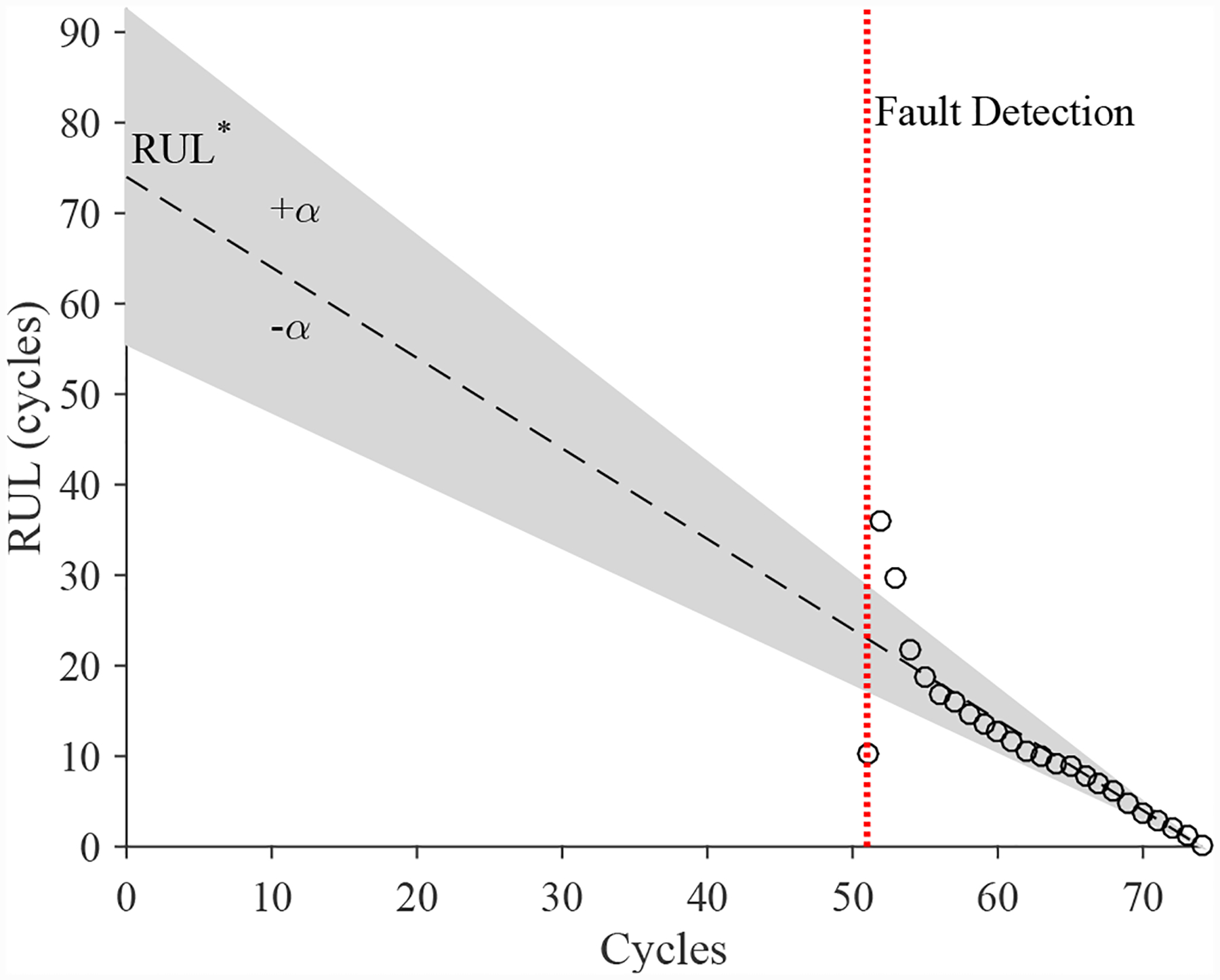
Predicted RUL values for the leak from supply for the DV.

The estimated leak parameter values, based on the open times of the DV, are shown in Fig. 22. In order to estimate the fault progression parameters, the last 15 values are used. The EOL predictions are given in Fig. 23 and the RUL values in Fig. 24, where *α* = 0.15 represents a desired accuracy constraint, EOL* denotes the true EOL, and RUL* denotes the true RUL. The predictions converge relatively quickly after the fault is detected, with *PH* = 13.04%. RA averages to 99.07%.

Over all experiments, PH averages to 14.83% and average RA to 98.22%. For this fault, the progression of the fault is relatively clear in the opening times, and so predictions are very accurate and are accurate early.

### 5.2. Continuous Valve

For CV leak from the signal line and the leak from the supply line faults are dicsussed.

#### 5.2.1. Leak from Signal Line

**Figure 25.**
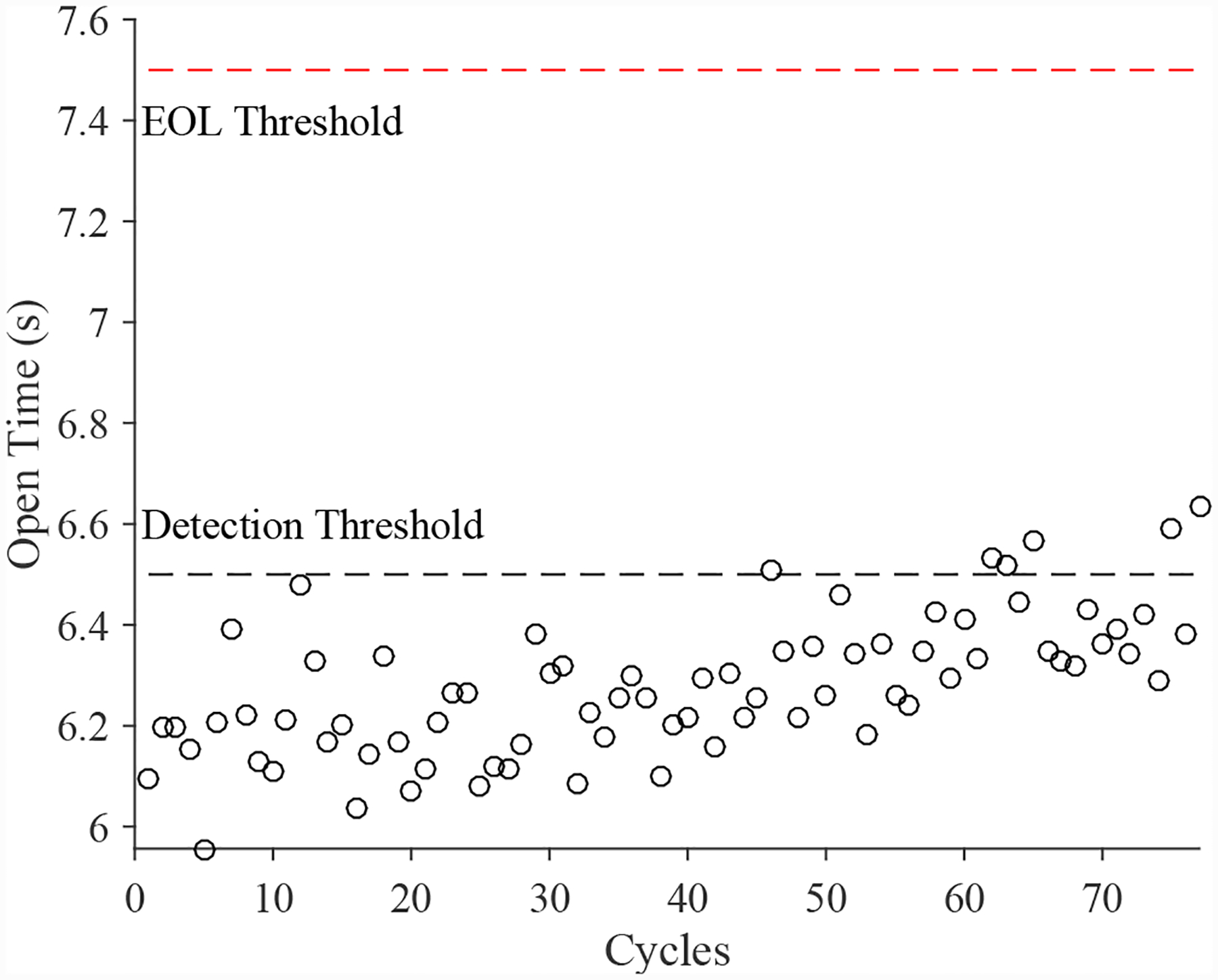
CV open times with a leak from signal line.

**Figure 26.**
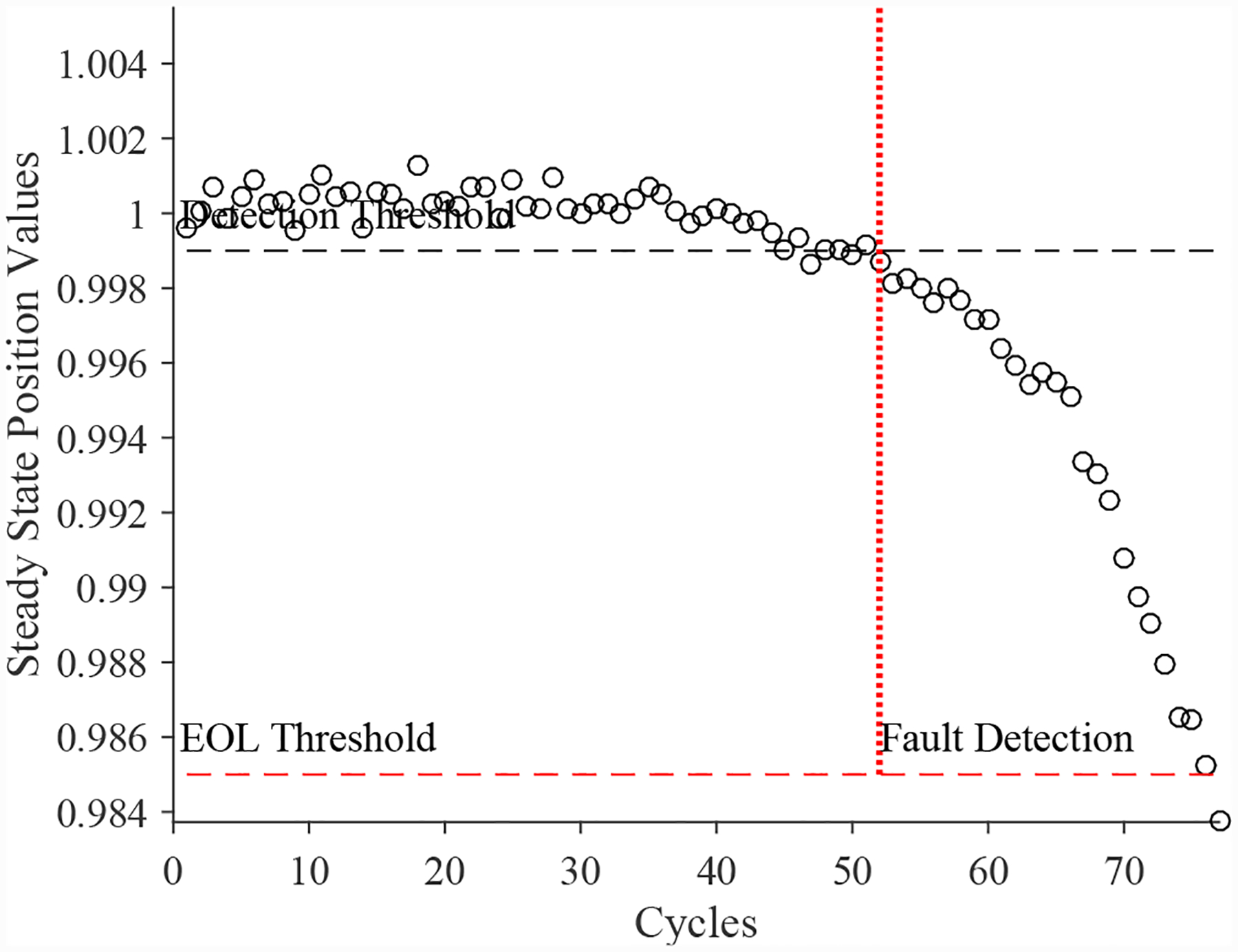
CV steady-state position with a leak from signal line.

A total of 4 experiments were performed for this fault. As described in Section 2, the leak from signal line fault is injected by controlling the position of the leak valve V3. As described in Section 3, this fault causes an increase in opening times and an increase in steady-state position error. Fig. 25 shows the open times of the valve during the fault progression, without a clear trend. Fig. 26 shows the steady-state position values, with a clear downward trend. A fault is detected at the 48th cycle based on the steady-state position.

**Figure 27.**
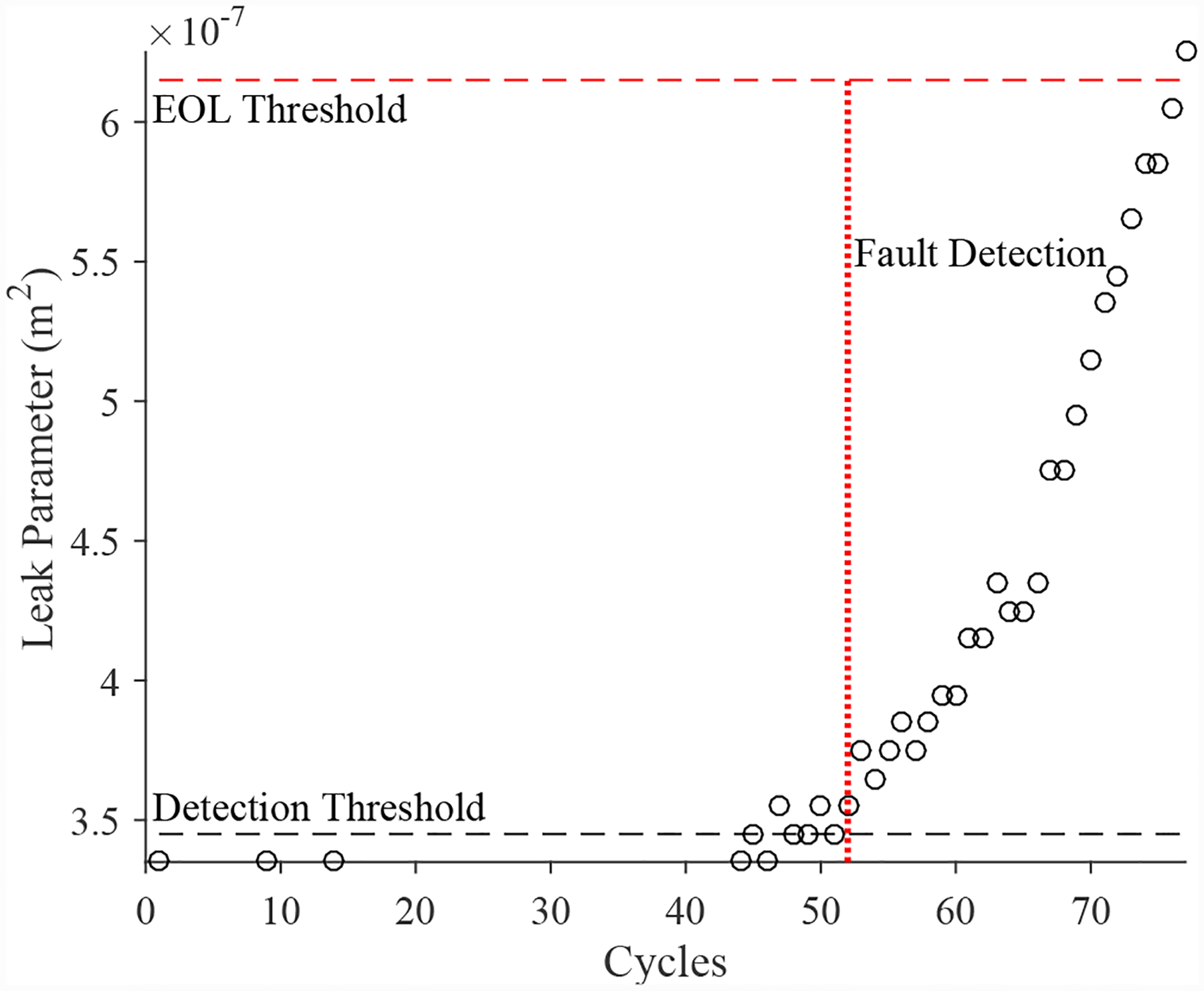
Estimated leak parameter values based on steady-state position for the leak from signal line for the CV.

**Figure 28.**
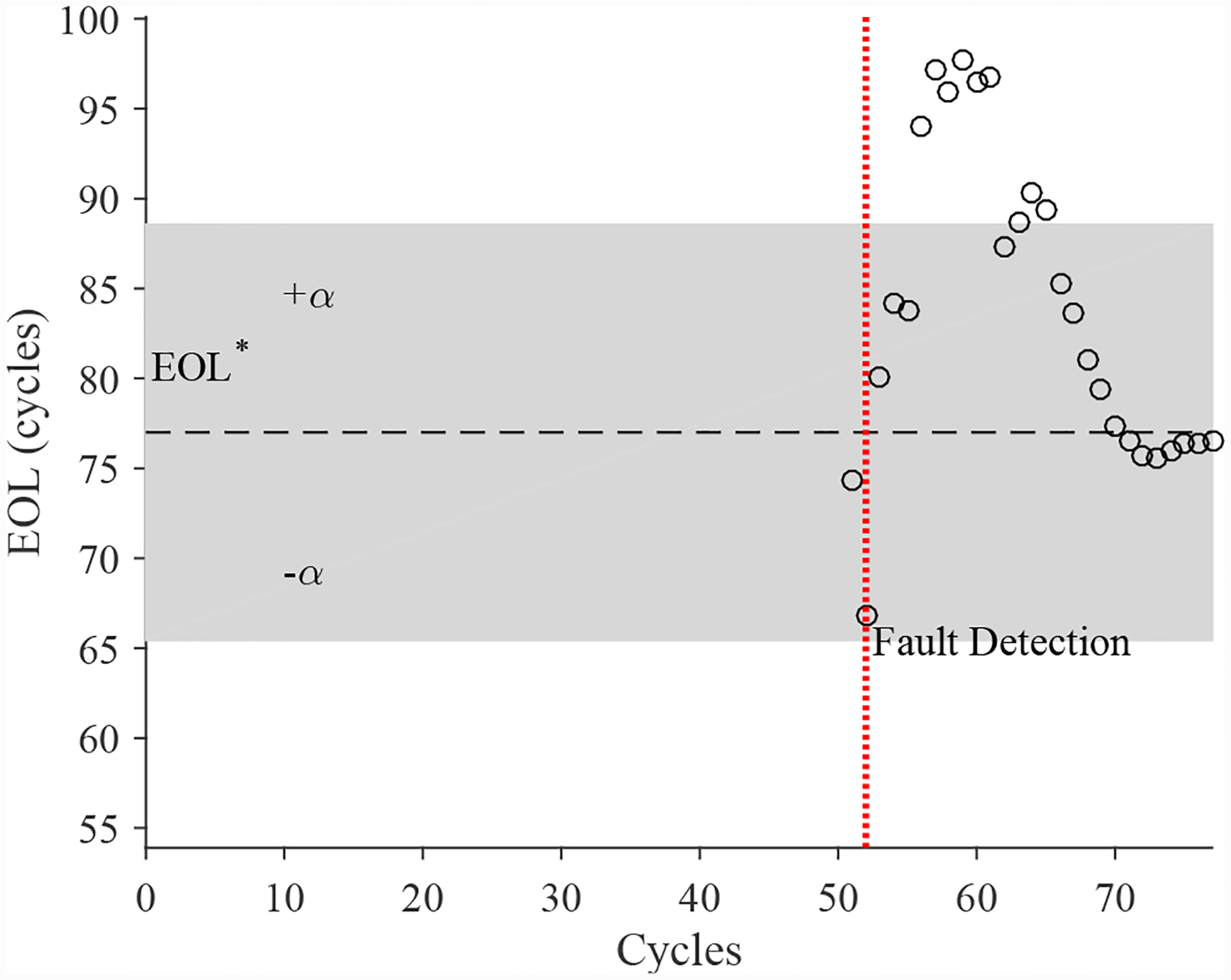
Predicted EOL values for the leak from signal line for the CV.

**Figure 29.**
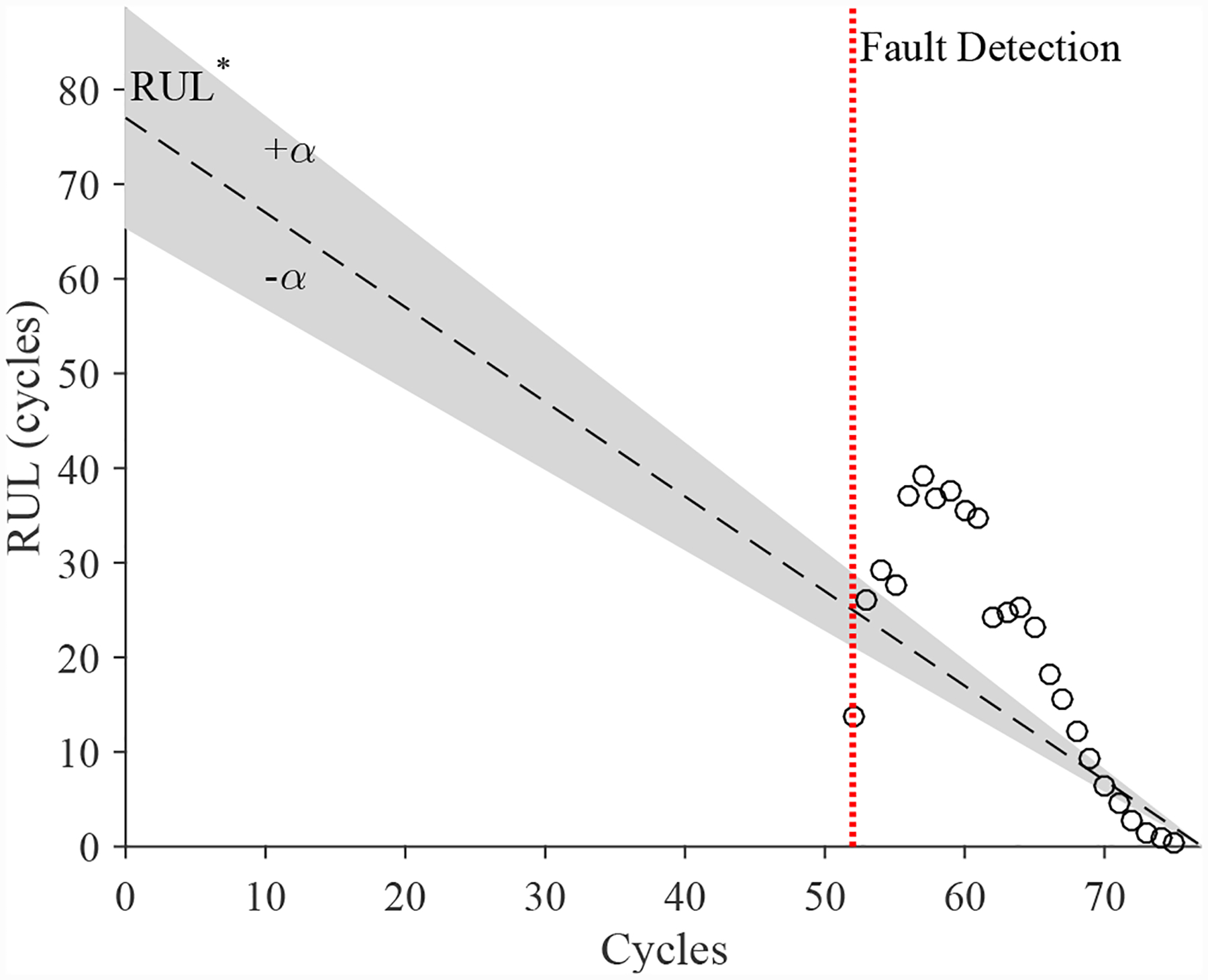
Predicted RUL values for the leak from signal line for the CV.

The estimated leak parameter values, based on the steady-state positions of the CV, are shown in Fig. 27. In order to estimate the fault progression parameters, the last 10 values are used. The EOL predictions are given in Fig. 28 and the RUL values in Fig. 29. The predictions converge more slowly than other faults, with *PH* = 60.00%. Due to the slower convergence, RA over the period from fault detection to EOL averages to 88.82%.

Results are similar for the other experiments. Over all 4 experiments, PH averages to 63.15%, and average RA to 83.90%.

#### 5.2.2. Leak from Supply Line

**Figure 30.**
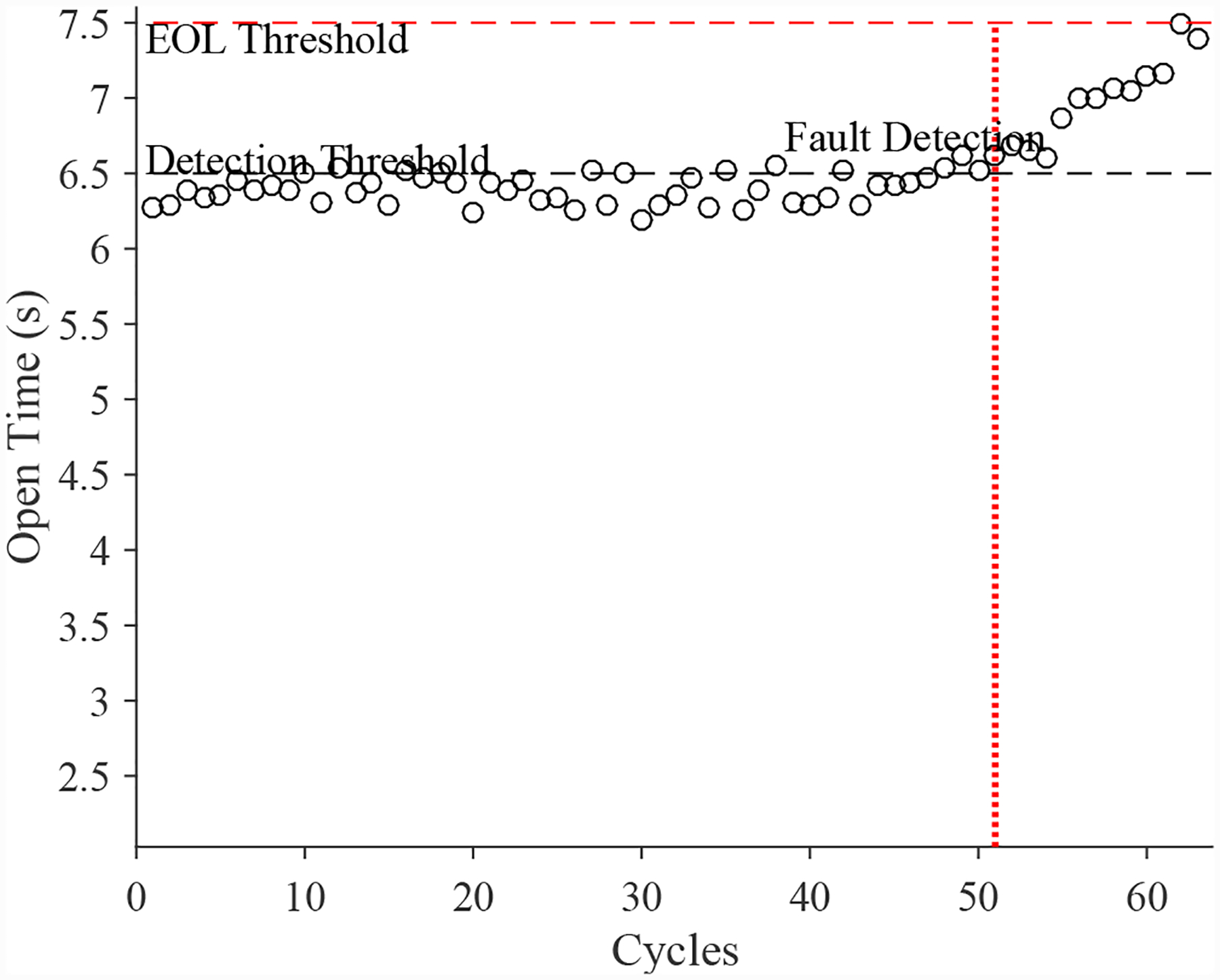
Valve open times with a leak from supply line for the CV.

A total of 6 experiments were performed for this fault. As described in Section 2, the leak from supply line fault is injected by controlling the position of the leak valve V4. As described in Section 3, this fault causes an increase in opening times and an increase in steady-state position error. Fig. 30 shows the open times of the valve during the fault progression, with a clear trend. Fig. 26 shows the steady-state position values, without a clear trend. A fault is detected at the 51st cycle based on the open times.

**Figure 32.**
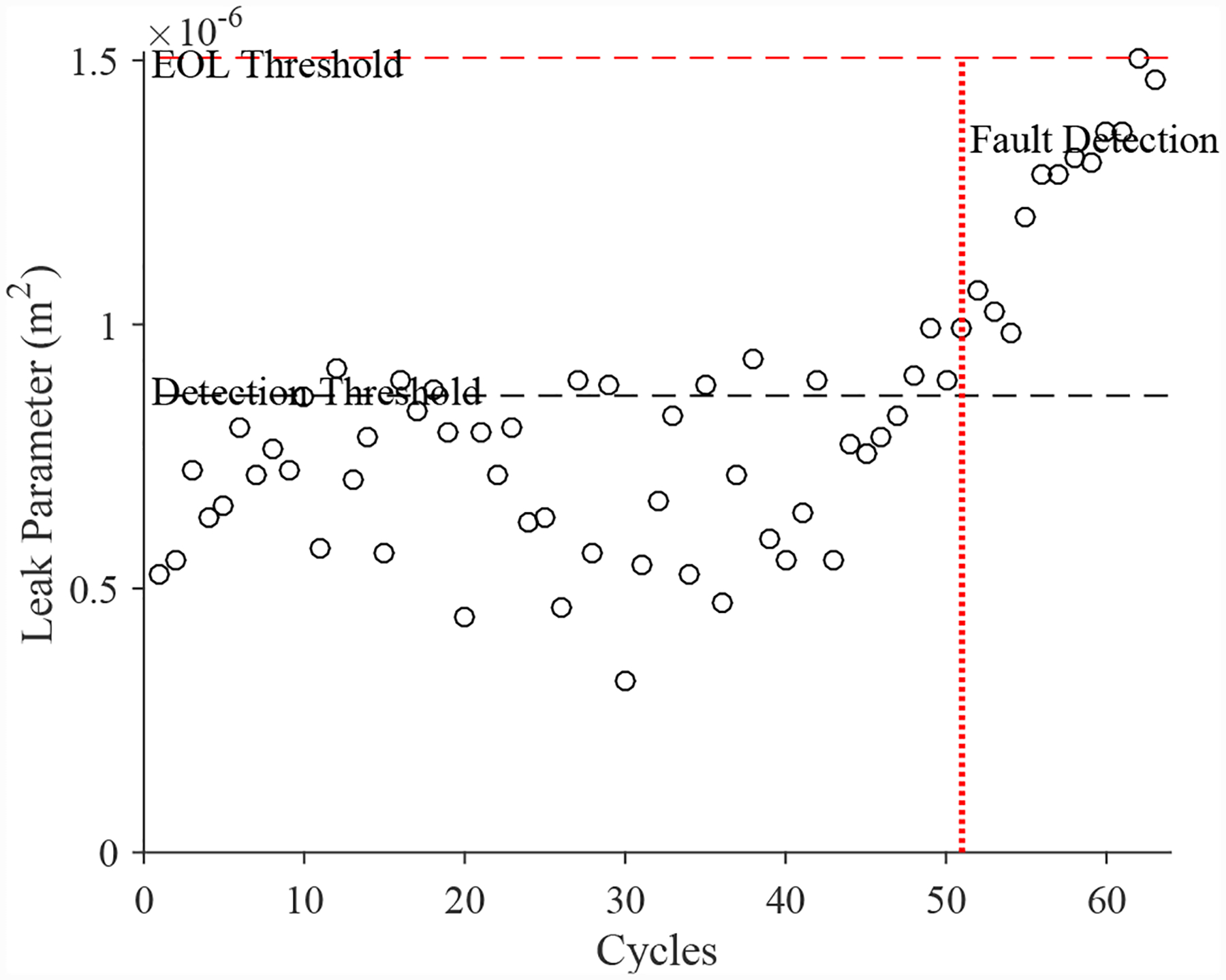
Estimated leak parameter values based on steady-state position for the leak from signal line for the CV.

**Figure 33.**
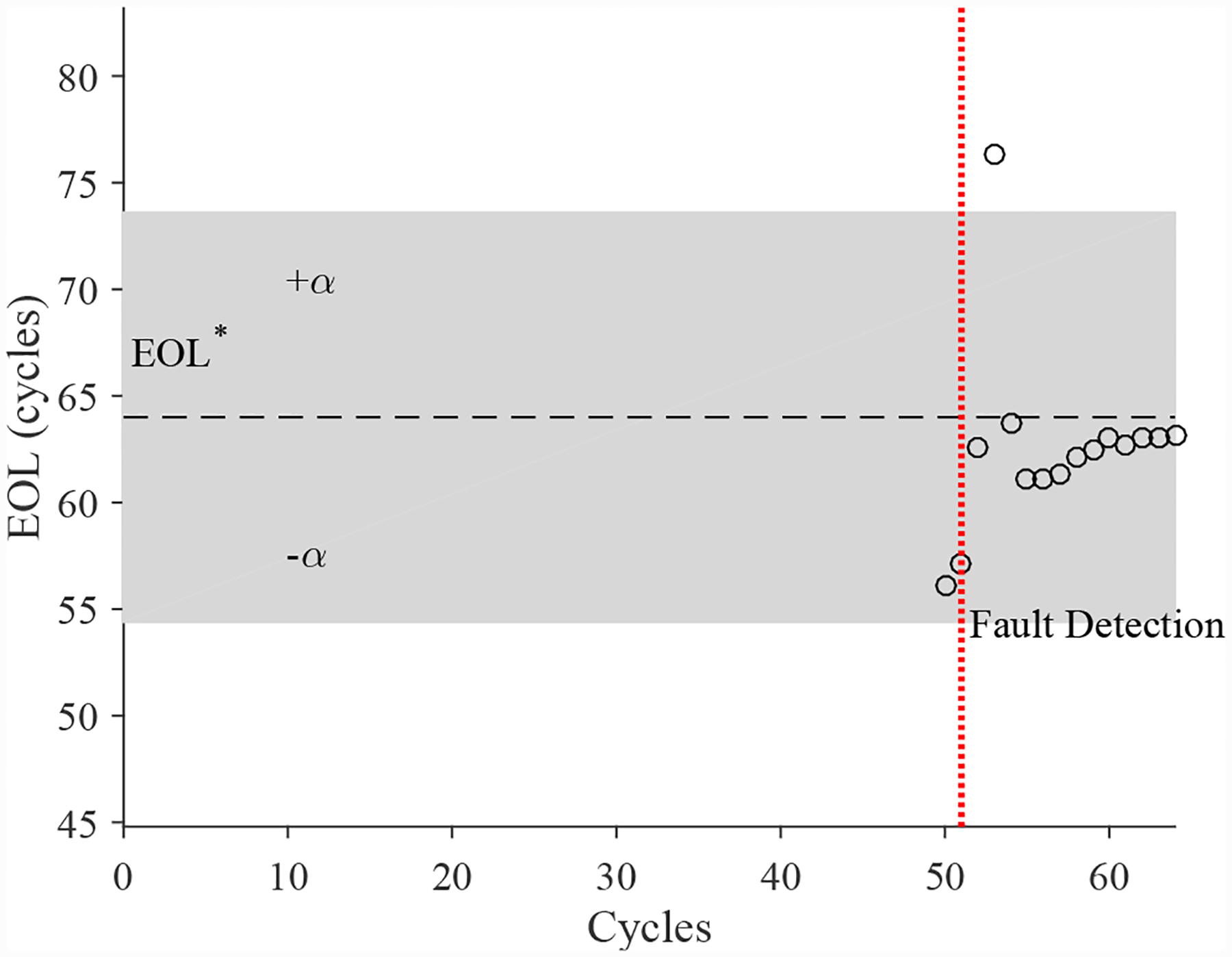
Predicted EOL values for the leak from supply line for the CV.

**Figure 34.**
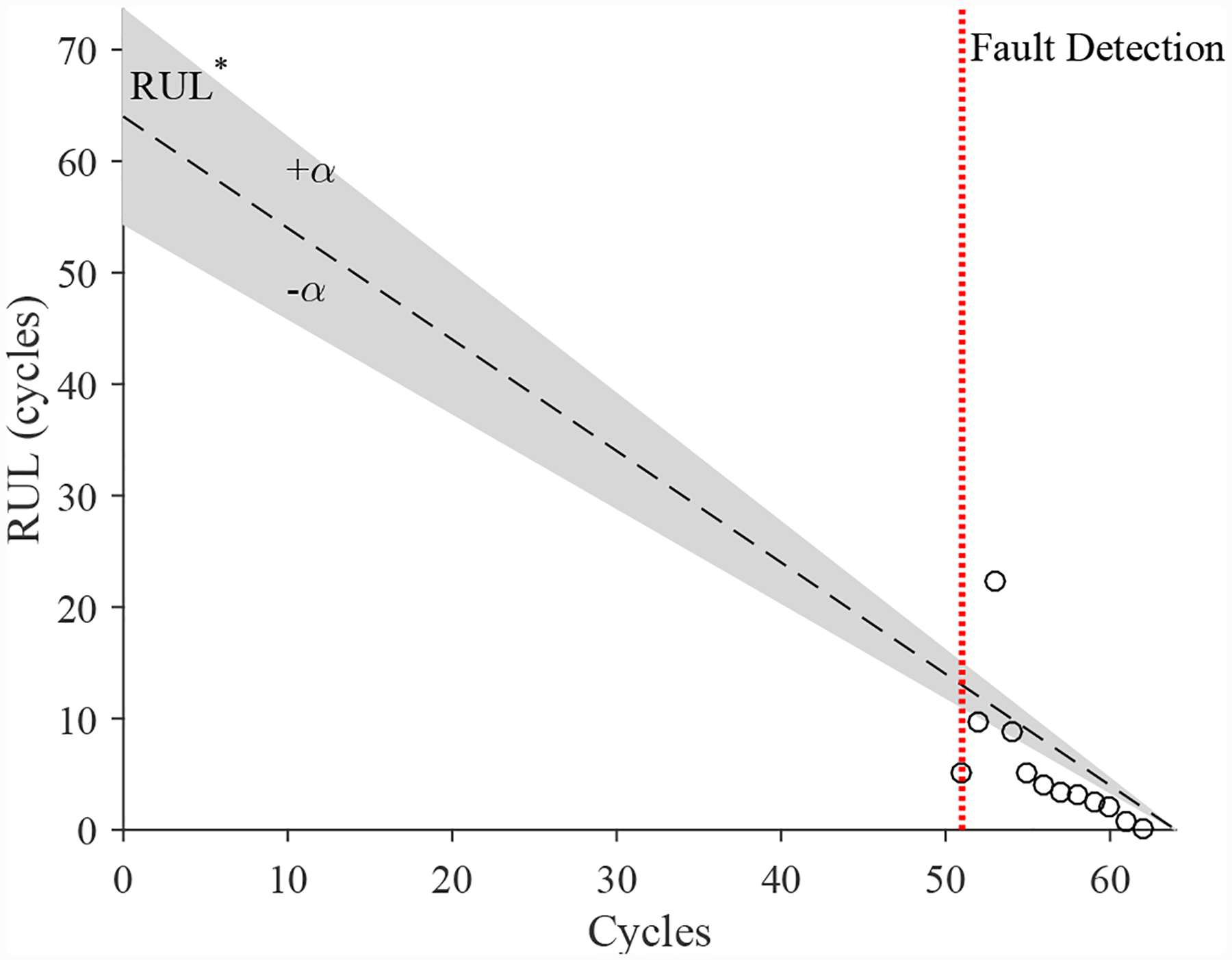
Predicted RUL values for the leak from supply line for the CV.

The estimated leak parameter values, based on the open times of the CV, are shown in Fig. 32. In order to estimate the fault progression parameters, all values are from the point of fault detection to the present time are used. The EOL predictions are given in Fig. 33 and the RUL values in Fig. 34. The predictions converge relatively quickly, with *PH* = 23.08%. RA averages to 97.54%.

Over all 6 experiments, PH averages to 29.85%, and average RA to 93.06%.

## 6. Related Work

Despite their prevalence in many domains, and their criticality in many kinds of system operations, applying prognostics to valves has only recently received attention in the scientific literature. In (Gomes, Ferreira, Cabral, Glavão, & Yoneyama, 2010), a valve in a pressure control system was investigated. The probability integral transform was used to compute a dissimilarity measure for the identification of anomalies and trends in anomalous behavior. However, no prediction method was developed.

The unscented particle filter is used by (Tao, Zhao, Zio, Li, & Sun, 2014) for the estimation of the health state of a pneumatic valve. Based on the predicted health distribution a replacement strategy is developed. The approach is validated only in simulation.

The prognostics of a launch valve in the steam catapult of an aircraft carrier is considered in (Shevach et al., 2014). A risk-sensitive particle filter is used for state estimation, and an exponential moving average filter is used for prediction. Like our approach, valve timing data is used for fault detection and as the basis for prediction. However, our approach predicts EOL/RUL based on a dynamic model, whereas this approach uses a trend learned from data with the moving average filter.

Pneumatic valves for air bleed systems in aircraft are considered in both (Lorton et al., 2013) and (Ribeiro, Yoneyama, Souto, & Turcio, 2015). In the former, a piecewise-deterministic Markov process (PDMP) modeling framework is used, with a Monte Carlo-based prediction approach. In the latter, only degradation level is identified and no prediciton is performed. A PDMP modeling framework with Monte Carlo-based prediction is also used in (Lin et al., 2014), but for a pneumatic valve in a nuclear power plant residual heat removal system.

## 7. Conclusions

This paper described development of a model-based prognostics approach to two types of pneumatic valves, for which a custom testbed provided run-to-failure data. The system health management functions exercised included fault detection, fault isolation, damage estimation, and remaining life prediction. The algorithms were validated on experimental results from the testbed, that allowed for faults to be injected and fault magnitude to be modulated according to a fault progression model. The function governing the fault progression model can be updated based on preferred fault propagation model choice.

Faults were detected late (with a prognostic horizon bar ~0.2) due to masking of the fault signatures in indirect sensor measurements (a fairly common problem in systems health management). Prediction after detection was quite accurate for the DV valve (with all predicted estimates falling within the 20% alpha cone at PH=0.28) but not as accurate for the CV valve (most values outside of alpha-lambda cone), owing to a convolution of sensor noise and model shortcomings. Nonetheless, the convergence performance was very high for all valves and fault modes

A limitation of the current approach is that the fault progression was carried out using a linear increase of the valve leakage. Although there is a nonlinear relationship between percent open and leak size/flow, this behavior does not necessarily represent the progression of a fault due to corrosion really well. A better model reflecting that relationship can be imposed on the testbed without any other change to the model of the valve or the testbed. Additional fault progression profiles representing other fault modes (besides corrosion) could easily be implemented since it would just be a change of the opening times of the proportional valves.

A CV valve would typically be opened to different positions. Whereas this information should be used (and possibly help to further improve performance), the work here only considered open/close information. Initial work in that direction for a rotary valve has been performed in (Daigle, 2015).

A further direction for future work is to consider uncertainty (Sankararaman, Daigle, & Goebel, 2014). Currently, uncertainty is ignored, although there is substantial uncertainty in the fault estimates and in the future valve operation, which can result in corresponding prediction uncertainty that should be captured. Another aspect to look into is correlating accelerated aging of the components with real life aging. Additional field usage data may help in mapping accelerated aging experimental data with real usage data.

## Figures and Tables

**Figure 31.**
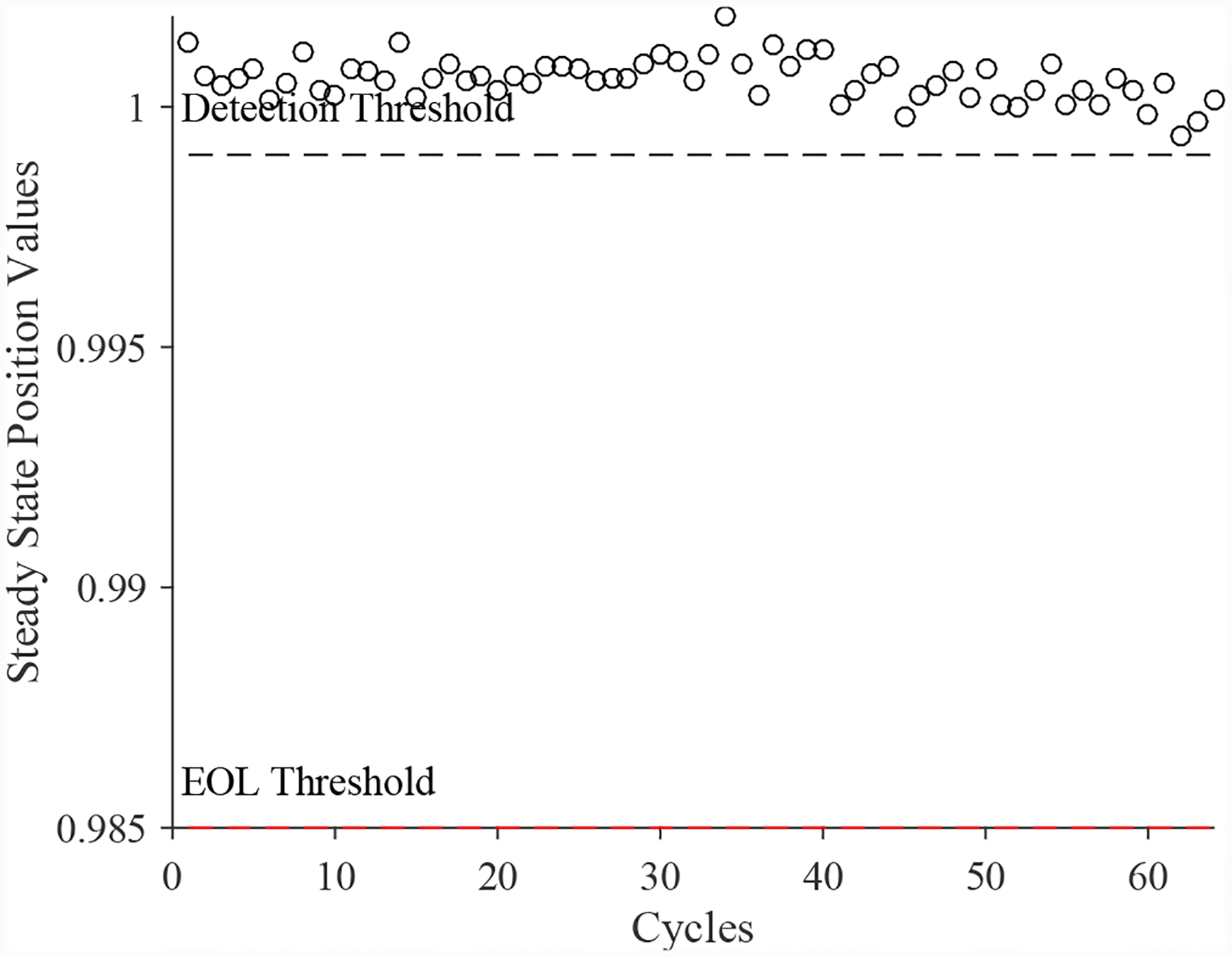
Valve steady-state position with a leak from supply line for the CV.

## Footnotes

1 Bold typeface denotes vectors, and *n*_*a*_ denotes the length of a vector **a**.
